# Effects of Exercise Training on Muscle Quality in Older Individuals: A Systematic Scoping Review with Meta-Analyses

**DOI:** 10.1186/s40798-023-00585-5

**Published:** 2023-06-06

**Authors:** Tibor Hortobágyi, Tomas Vetrovsky, Jennifer S. Brach, Martijn van Haren, Krystof Volesky, Regis Radaelli, Pedro Lopez, Urs Granacher

**Affiliations:** 1 Department of Kinesiology, Hungarian University of Sports Science, Budapest, Hungary; 2 Institute of Sport Sciences and Physical Education, University of Pécs, Pecs, Hungary; 3 Somogy County Kaposi Mór Teaching Hospital, Kaposvár, Hungary; 4 Center for Human Movement Sciences, University of Groningen Medical Center, Groningen, The Netherlands; 5 Institute of Sport Research, Sports University of Tirana, Tirana, Albania; 6 Faculty of Physical Education and Sport, Charles University, Prague, Czech Republic; 7 Department of Physical Therapy, University of Pittsburgh, Pittsburgh, PA USA; 8 Faculty of Human Kinetics, CIPER, University of Lisboa, Cruz Quebrada, Dafundo, Portugal; 9 Exercise Medicine Research Institute, Edith Cowan University, Joondalup, Australia; 10 School of Medical and Health Sciences, Edith Cowan University, Joondalup, WA Australia; 11 Department of Sport and Sport Science, Exercise and Human Movement Science, University of Freiburg, Freiburg, Germany

**Keywords:** Aging, Neurological disease, Resistance training, Muscle mass, Intramuscular fat

## Abstract

**Background:**

The quantity and quality of skeletal muscle are important determinants of daily function and metabolic health. Various forms of physical exercise can improve muscle function, but this effect can be inconsistent and has not been systematically examined across the health-neurological disease continuum. The purpose of this systematic scoping review with meta-analyses was to determine the effects and potential moderators of exercise training on morphological and neuromuscular muscle quality (MMQ, NMQ) in healthy older individuals. In addition and in the form of a scoping review, we examined the effects of exercise training on NMQ and MMQ in individuals with neurological conditions.

**Methods:**

A systematic literature search was performed in the electronic databases Medline, Embase, and Web of Science. Randomized controlled trials were included that examined the effects of exercise training on muscle quality (MQ) in older individuals with and without neurological conditions. Risk of bias and study quality were assessed (Cochrane Risk of Bias Tool 2.0). We performed random-effects models using robust variance estimation and tested moderators using the approximate Hotelling–Zhang test.

**Results:**

Thirty studies (*n* = 1494, 34% females) in healthy older individuals and no studies in individuals with neurological conditions were eligible for inclusion. Exercise training had small effects on MMQ (*g* = 0.21, 95% confidence interval [CI]: 0.03–0.40, *p* = 0.029). Heterogeneity was low (median *I*^2^ = 16%). Training and demographic variables did not moderate the effects of exercise on MMQ. There was no association between changes in MMQ and changes in functional outcomes. Exercise training improved NMQ (*g* = 0.68, 95% CI 0.35–1.01, *p* < 0.000) across all studies, in particular in higher-functioning older individuals (*g* = 0.72, 95% CI 0.38–1.06, *p* < 0.001), in lower extremity muscles (*g* = 0.74, 95% CI 0.35–1.13, *p* = 0.001), and after resistance training (*g* = 0.91; 95% CI 0.42–1.41, *p* = 0.001). Heterogeneity was very high (median *I*^2^ = 79%). Of the training and demographic variables, only resistance training moderated the exercise-effects on NMQ. High- versus low-intensity exercise moderated the exercise-effects on NMQ, but these effects were considered unreliable due to a low number of studies at high intensity. There was no association between changes in NMQ and changes in functional outcomes.

**Conclusion:**

Exercise training has small effects on MMQ and medium-large effects on NMQ in healthy older individuals. There was no association between improvements in MQ and increases in muscle strength, mobility, and balance. Information on dose-response relations following training is currently lacking. There is a critical gap in muscle quality data for older individuals with lower function and neurological conditions after exercise training. Health practitioners should use resistance training to improve muscle function in older individuals. Well-designed studies are needed to examine the relevance of exercise training-induced changes in MQ in daily function in older individuals, especially to those with lower function and neurological conditions.

**Supplementary Information:**

The online version contains supplementary material available at 10.1186/s40798-023-00585-5.

## Key points

- The quantity and quality of skeletal muscle are important determinants of daily function and metabolic health
- Exercise training had small effects on morphological muscle quality (MMQ) in healthy older adults but improved neuromuscular muscle quality (NMQ) with a medium-to-large effect size
- Resistance training versus other exercise interventions moderated the exercise effects on NMQ
- High- versus low-intensity exercise moderated the exercise effects on NMQ but these effects are considered unreliable due to a low number of studies at high intensity
- Improvements in NMQ were not associated with improvements in mobility or balance
- Health practitioners should use resistance training to improve muscle function in older individuals

## Introduction

Skeletal muscle forms ~ 40% of human body mass [1, 2]. The quantity of muscle varies greatly among individuals with 35–50 kg in young men and can be as low as ≤ 13 kg in elderly women [3]. With natural aging, muscle mass starts to decline at an age zone of 35–50 years, depending on health status, sex, muscle group, and measurement method [2, 4–10]. Low muscle mass is associated with a reduced muscle strength (i.e., dynapenia) [11–14], fitness [15], mobility [14], postural stability [16], release of anti-inflammatory myokines [17], brain gray matter volume [18], therapeutic efficacy [19], and quality of life [20, 21]. On the other hand, low age-related muscle mass (i.e., sarcopenia) is associated with an increase in falls [22–24], frailty [25], fractures [26], intra- and inter-muscular fat accumulation [27, 28], risks for cardiovascular disease [29], incidence of cancer [30–32], mental health problems [20, 33], cognitive impairment [34], hospitalization [24], morbidity, and mortality [35–41].

Age-related decline in physical activity exacerbates muscle atrophy and weakness [42]. Muscle mass loss coupled with low muscle strength and reduced physical function constitute sarcopenia [1], a condition now with an international disease classification code [43]. Prevalence of sarcopenia gave rise to a geriatric pandemic [25], making ‘Musclespan’ a key component of ‘Healthspan’. Minimizing age- and disease-related muscle loss has become a public health priority.

Muscle quality (MQ) further specifies the above-described changes in whole-body and regional skeletal muscle loss in older individuals and clinical conditions. Morphological MQ (MMQ) measures non-contractile (intermuscular, intramuscular adipose and fibrous) tissue in absolute units and relative to total limb muscle size using imaging methods [44–49]. High quantities of such tissues have unfavorable effects on physical function and metabolic health [44, 45]. Neuromuscular MQ (NMQ) is the ratio between the force or torque generated or a load lifted relative to muscle thickness, volume, cross-sectional area or echo intensity [44, 47, 48]. While MMQ expresses MQ as the quantity of mechanical, architectural, and metabolically active tissue compartments, NMQ expresses the functionality of the muscle tissue with respect to force generation [44].

Physical exercise has beneficial effects on physical fitness, metabolic, and inflammatory processes across the lifespan [50] and is a favorable modifier of MMQ and NMQ [23, 25, 28, 51, 52]. However, it is unclear if the MQ-promoting effects of physical exercise differ along the health-disease continuum. For example, while resistance training, an anabolic exercise stimulus, improved functional outcomes and MQ [53–55], these improvements were small and occurred independent of training variables [28]. In addition, the effects of exercise training on MQ are also unclear in patients with neurological conditions such as Parkinson’s disease [56–58], multiple sclerosis [59–63], and stroke [64–71]. In individuals with neurological conditions, the disease amplifies age-related muscle loss [72] yet it remains unexamined to what extent exercise training could counteract sarcopenia in these patients. As is the case with dynapenia and functional capacity [73], sarcopenia is also greater in lower compared with upper extremity muscles [74, 75]. Whether sarcopenia and subsequent exercise training each impact upper and lower extremity muscles similarly in older individuals with and without neurological conditions has not yet been systematically examined [76–81]. Additionally, because aging and disease tend to reduce responsiveness to the exercise stimulus, dietary supplements are often added to exercise training [44, 67, 81–84]. However, it is unclear if, compared with exercise training-only, combining dietary supplements with exercise training would produce greater effects on MQ in older individuals with and without neurological conditions.

Therefore, the purpose of this systematic scoping review with meta-analyses was to determine the effects and potential moderators of exercise training on morphological and neuromuscular muscle quality (MMQ, NMQ) in lower- and higher-functioning healthy older individuals, a comparison that is new. We identified lower versus higher-functioning older individuals based on walking speed (< 1.0 m/s), short physical battery performance score (≤ 8.0) and author-reported diagnosis of sarcopenia. In addition and in the form of a scoping review, we examined for the first time the effects of exercise training on NMQ and MMQ in individuals with neurological conditions. We addressed the following questions: 1) what are the exercise training effects on MQ and do these effects differ between low and high-functioning older individuals and those with neurological conditions?; 2) which demographic, training variables, and MQ measurement methods are associated with changes in measures of MQ?, and 3) which variables moderate the exercise-training effects on MQ? Answers to these questions would expand and further synthesize our current understanding of exercise training-improved MQ’s role in older adults’ physical function [27, 28, 44, 46, 49, 60, 67, 74, 85].

## Methods

### Literature Search and Selection Criteria

**Fig. 1:**
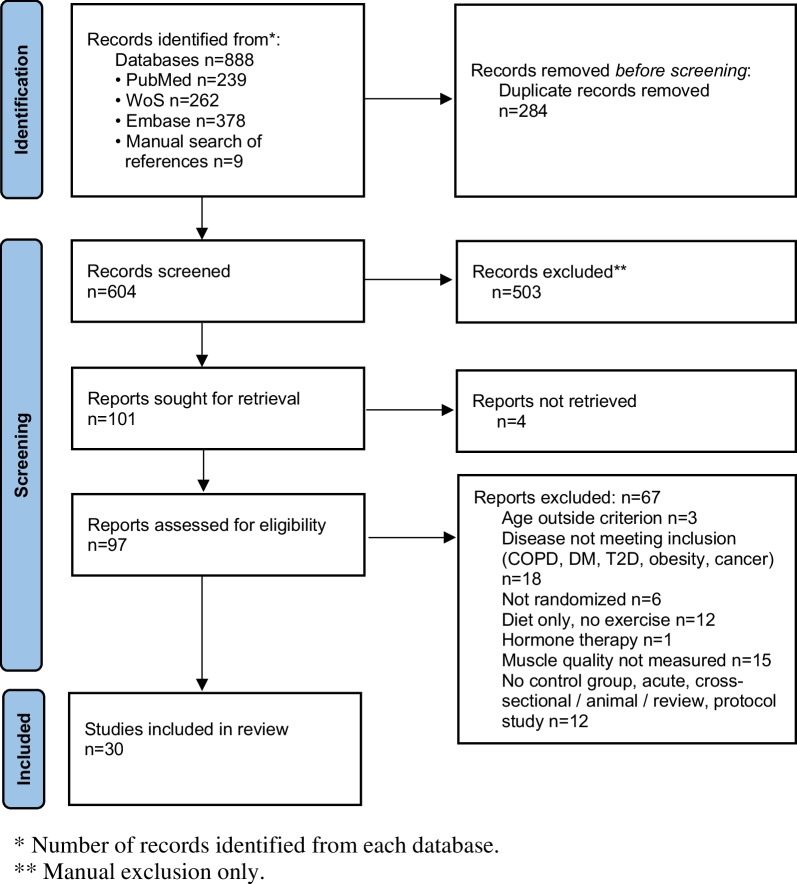
PRISMA flowchart. WoS, Web of Science; COPD, chronic obstructive pulmonary disease; DM, diabetes mellitus; T2D, type 2 diabetes mellitus

A library expert designed the search syntaxes (Additional file 1). Figure 1 shows the PRISMA flowchart. Inclusion and exclusion criteria were defined according to the population, intervention, comparators, outcomes, and study design criteria (Table 1). Concerning the population category, studies were eligible when including either higher- or lower-functioning healthy older individuals aged ≥ 65 years. We determined the age criterion by averaging the age of participants across the training and control groups in each study and compared it against the age limit of 65 years. For lower-functioning older individuals, we used walking speed (< 1.0 m/s), short physical battery performance score (≤ 8.0) and author-reported diagnosis of sarcopenia [86, 87]. Individuals with neurological conditions (e.g., Parkinson’s disease, multiple sclerosis, stroke) were included without restrictions of age, medication, disease stage, or disease sub-type and were included based on the author-reported diagnosis criteria. Concerning exercise training, we included resistance training, aerobic training, multicomponent training, and other single-mode exercise interventions (e.g., aquatic, dance exercise) with or without dietary supplements. We considered both active (i.e., contrast with different training programs) and passive, placebo or wait-list control groups as comparators. MMQ outcomes included intermuscular and intramuscular adipose and fibrous tissue, i.e., the amount of non-contractile tissue expressed in absolute terms and relative to total muscle size determined by imaging (e.g., magnetic resonance imaging [MRI], peripheral quantitative computed tomography [pQCT], computed tomography [CT], or ultrasound imaging [US]). NMQ outcomes were the ratio of maximal isometric or dynamic voluntary force, torque, or the load lifted relative to muscle size, i.e., muscle thickness, muscle mass, muscle volume or muscle cross-sectional area determined by MRI, pQCT, CT, US and also by dual-energy X-ray absorptiometry (DXA). We included both upper and lower extremity muscles. We included only randomized controlled trials. By including upper extremity and dietary supplement data, we modified the registered review to increase scope.

**Table 1:** Selection criteria according to PICOS criteria (population, intervention, comparator, outcome, study design

Category	Inclusion criteria	Exclusion criteria
Population	Higher- or lower-functioning healthy older individuals age ≥ 65 years or individuals with neurological conditions (e.g., Parkinson’s disease, multiple sclerosis, stroke) We determined the age criterion by averaging the age of participants across the training and control groups in each study and compared it against the age limit of 65 years. For lower-functioning older individuals, we used walking speed (< 1.0 m/s), short physical battery performance score (≤ 8.0) and author-reported diagnosis of sarcopenia	Higher- or lower-functioning healthy older individuals age < 65 years or individuals with neurological conditions (e.g., Parkinson’s disease, multiple sclerosis, stroke)
Intervention	Resistance training, aerobic training, multimodal training, and other single-mode exercise interventions (e.g., aquatic, dance exercise) with or without dietary supplements	Studies that were shorter than six weeks in duration or used fewer than nine exercise sessions
Comparator	Active (i.e., contrast with different training programs) and passive, placebo control or wait-list control groups	No control group
Outcome	MMQ outcomes included intermuscular and intramuscular adipose and fibrous tissue, i.e., the amount of non-contractile tissue expressed in absolute terms and relative to total muscle size determined by imaging (e.g., magnetic resonance imaging [MRI], peripheral quantitative computed tomography [pQCT], computed tomography [CT], or ultrasound imaging [US]). NMQ outcomes were the ratio of maximal isometric or dynamic voluntary force, torque, or the load lifted relative to muscle size, i.e., muscle thickness, muscle mass, muscle volume or muscle cross-sectional area determined by MRI, pQCT, CT, US and also by dual-energy X-ray absorptiometry (DXA). We included both upper and lower extremity muscles	Failed to report the pre-post means or change scores and their standard deviations numerically or graphically; studies for which authors did not reply to our inquiries sent by email; failed to use at least one marker of MMQ or NMQ
Study design	Randomized controlled trials including upper extremity and dietary supplement data	Case report, used a cross-over design or a diet-only intervention

MMQ, NMQ: morphological and neuromuscular muscle quality

Studies were excluded when: 1) it was clear from the article title or abstract that the trial was not relevant or if it did not meet inclusion criteria; 2) the journal was not peer-reviewed (e.g., gray literature); 3) the trial failed to use at least one marker of MMQ or NMQ; 4) the trial failed to report the pre-post means or change scores and their standard deviations numerically or graphically; and 5) the article was a case report, used a cross-over design or a diet-only intervention. To maximize muscular versus neural adaptations, we excluded studies that were shorter than six weeks in duration or used fewer than nine exercise sessions. While specific cut-offs of exercise intervention duration and total number of sessions have not yet been established with respect to MQ [88, 89] and intervention duration was not associated with MQ [28], we used these intervention duration and session number cut-offs in an anticipation to induce robust instead of transient changes in measures of muscle quality. Also, a previous systematic review with meta-analysis reported that ‘Meta-regression of data from 25 studies revealed that a resistance training program with the goal to increase healthy old adults’ muscle strength is characterized by a training period of 50–53 weeks…’, suggesting the inclusion of studies with longer intervention duration and higher number of session number is likely to improve MQ [90]. Searches were limited to human studies reported in full-text articles in English.

Two authors, blinded to each other, independently screened the titles and abstracts of the records resulting from database searches. After this initial screening, the two authors unblinded their decisions and disagreements were resolved through discussions with a third author. Reference lists of the included studies and relevant review articles were screened for the inclusion of additional potential studies. Duplicates between searches were removed.

### Data Extraction

We extracted the following information from each study: first author, publication year, population (higher- and lower-functioning older individuals, individuals with neurological conditions), age, sex, participant number, type of exercise intervention (resistance, aerobic, multimodal, other), dietary supplement use, intervention duration, total number of training sessions, session duration, exercise intensity (low, high), limb (arm, leg), MQ outcomes (MMQ, NMQ), and functional outcomes (mobility, strength/power, balance). In several cases, we requested data from corresponding authors and if not received, the study was excluded. When data were reported only in charts, a semi-automated software (https://automeris.io/WebPlotDigitizer/) was used to extract data from *x*–*y* plots. Low versus high intensity was determined for aerobic training based on the percentage of maximal oxygen uptake (with 63% as the threshold between the two intensities), percentage of maximal heart rate (77%), heart-rate reserve (60%), metabolic equivalent (6.0), rate of perceived exertion (Borg scales 6–20: 15/20; 0–10: 5/10), second ventilatory threshold, and anaerobic threshold (below: low intensity) [91, 92]. Intensity for resistance training was categorized as low intensity versus high when the load was, on average, below 69% of 1-repetition maximum and high when, on average, the load was above 69% of the one-repetition maximum [91, 92]. For multicomponent and other training, the training parameters were also determined based on the above or other reported training descriptors (elastic band color coding, dumbbell weights, vibration frequency, heart rate during dancing). When aerobic and resistance training intensity progressively increased during an intervention, we averaged the intensity values to arrive at an overall intervention intensity. Two authors blinded to each other independently performed these categorizations and disagreements were resolved by consensus. If a study had two groups performing different interventions, these intervention arms were coded as a separate study.

Measures of muscle density, echo intensity, and intramuscular fat were categorized as MMQ, with a positive (density, high density) or negative sign (intramuscular fat, low density area, echo intensity) for improvement [28]. Measures of force, torque, and load expressed relative to muscle thickness, muscle mass, muscle volume or muscle cross-sectional area were coded as NMQ. Functional outcomes were coded as: mobility (walking speed/time/distance, timed up and go test under single and cognitive dual task conditions); muscle strength/power (maximal voluntary contraction of handgrip, shoulder, wrist, elbow, hip, knee, ankle muscle groups, stair climbing, jumping, object carrying, chair rise time, number), and balance (standing time, postural sway, tandem gait).

### Risk of Bias Assessment

The risk of bias was evaluated according to the second version of the Cochrane risk-of-bias tool for randomized trials. Each assessment focused on the outcome level [93]. The five-domain instrument includes: 1) randomization process; 2) deviation from intended interventions; 3) missing outcome data; 4) measurement of the outcome; 5) selection of the reported result, and 6) overall bias (summary score, derived from the 5 items). Overall risk of bias was expressed as “low risk of bias” if all domains were classified as low risk, “some concerns” if some concern was raised in at least one domain but not classified as at high risk in any other, or “high risk of bias” if at least one domain was classified as high risk, or multiple domains had some concerns.

### Statistical Analyses

We used robumeta (version 2.0), metafor (version 3.4-0), and clubSandwich (version 0.5.6) packages in R, version 4.2.0 (The R Foundation for Statistical Computing, Vienna, Austria). To pool the data, a robust variance estimation random-effects meta-analytical approach was applied which allows for the inclusion of multiple dependent outcomes from the same study. Robust variance estimation assesses the variance of regression coefficient estimates with the use of the observed residuals and does not require the weights or distributional assumptions [94, 95]. To account for the correlated effects within the studies, ‘study’ was used as the clustering variable.

First, we computed the overall summary effects of all included studies separately for MMQ and NMQ and visualized them using forest plots. Sensitivity analyses were undertaken by assessing the effects of influential cases on the results. The influential cases were diagnosed using a combination of several methods (externally standardized residuals, difference in fit values, Cook's distances, covariance ratios, leave-one-out estimates of the amount of heterogeneity, leave-one-out values of the test statistics for heterogeneity, hat values, weights) as implemented in the 'influence' function within the metafor package [96]. We also examined potential publication bias using a funnel plot and Egger's regression test [97].

Second, we performed a series of subgroup meta-analyses for different populations, exercise types, training intensities, muscle groups and limbs (upper, lower), presence of dietary supplementation, use of active versus passive control group, methods of determination and measures of the MQ outcomes. Exercise types included resistance training and 'other exercise interventions', which combined aerobic, multimodal, and other training interventions due to their low yield. For all analyses, we computed Hedge’s g effect size (*g* = 0.15, 0.40, 0.75, respectively, small, medium and large effects) [98], standard error (SE) of the effect size, 95% confidence intervals, the statistical significance of the effect (set at *p* < 0.05), and heterogeneity statistics *I*^2^ and Tau^2^. The values of *I*^2^ > 25%, > 50%, and > 75% indicated, respectively, low, moderate, and high heterogeneity [99]. The direction of effect sizes was standardized so that positive effect sizes would reflect improvements in a given outcome.

Third, to explore potential effect moderators, we constructed full meta-regression models (separately for MMQ and NMQ) including age, sex, population, exercise type, exercise intensity, muscle group, limb, presence of dietary supplementation, method of determination of the MQ outcome, intervention duration, the total number of sessions, session duration, whether the study used an active or passive control group, and their interactions as covariates. Potential moderators and their interactions were removed from the full model one-by-one when not significant (*p* > 0.05), resulting in a final model where only those with significant moderating effects were included. The statistical significance of categorical moderators with three or more levels was tested using the Hotelling–Zhang test.

Finally, to assess the association between MQ and functional outcomes within individual studies, a series of random-effects meta-regression models were undertaken treating the MQ effect sizes as the outcome variables and the functional outcome effect sizes as predictor variables [100]. These association analyses examined if exercise-induced changes in MQ were related to changes in functional outcomes (i.e., walking speed, balance, muscle strength). Because the current review was anchored on MQ, we did not perform meta-analyses on functional outcomes. Such analyses would have yielded biased effect size estimates, as we would have failed to include potentially hundreds of studies summarized by targeted reviews of the effects of exercise on functional outcomes.

## Results

### Characteristics of the Included Studies

The search identified 888 records as of 1 April 2023. With duplicates removed, we screened 604 records and identified 30 studies for inclusion (Fig. 1) [10, 54, 76–81, 83–85, 101–119]. Of these, we coded 23 studies examining high-functioning and 7 studies examining low-functioning older individuals, and 0 studies examining individuals with a neurological condition.

A total of 1,494 older individuals (34% female), with a median age of 71 years (1st and 3rd interquartile range, IQR: 67–77) were enrolled in the 30 studies. Seven studies used dietary supplements. The median intervention duration was 12 weeks (IQR: 12–24), with a median of 36 exercise sessions scheduled (IQR: 24–48) for a median session duration of 50 min (IQR:40–60) at a median frequency of 2/week (IQR:2–3).

Six and 24 studies used, respectively, high and low exercise intensities, contrasting these effects against passive (*n* = 22 studies) or an active control group (*n* = 8; stretching, walking, health/diet education, diet placebo, cognitive exercise). MMQ was determined by CT, DXA, MRI, and pQCT (*n* = 13 studies). Measures of torque, force, or load were expressed relative to muscle thickness, cross-sectional area, muscle volume, and muscle mass to derive NMQ (*n* = 21 studies). Four studies reported measures for both MMQ and NMQ. MQ data were reported for muscles in the trunk only (*n* = 1 study), upper extremity only (*n* = 1 study), lower extremity only (*n* = 19 studies), or in both lower and upper extremities (*n* = 9 studies). Additional file 1: Table S1 shows the study characteristics.

### Overall Effects and Subgroup Analyses of Exercise Effects on MMQ

**Fig. 2:**
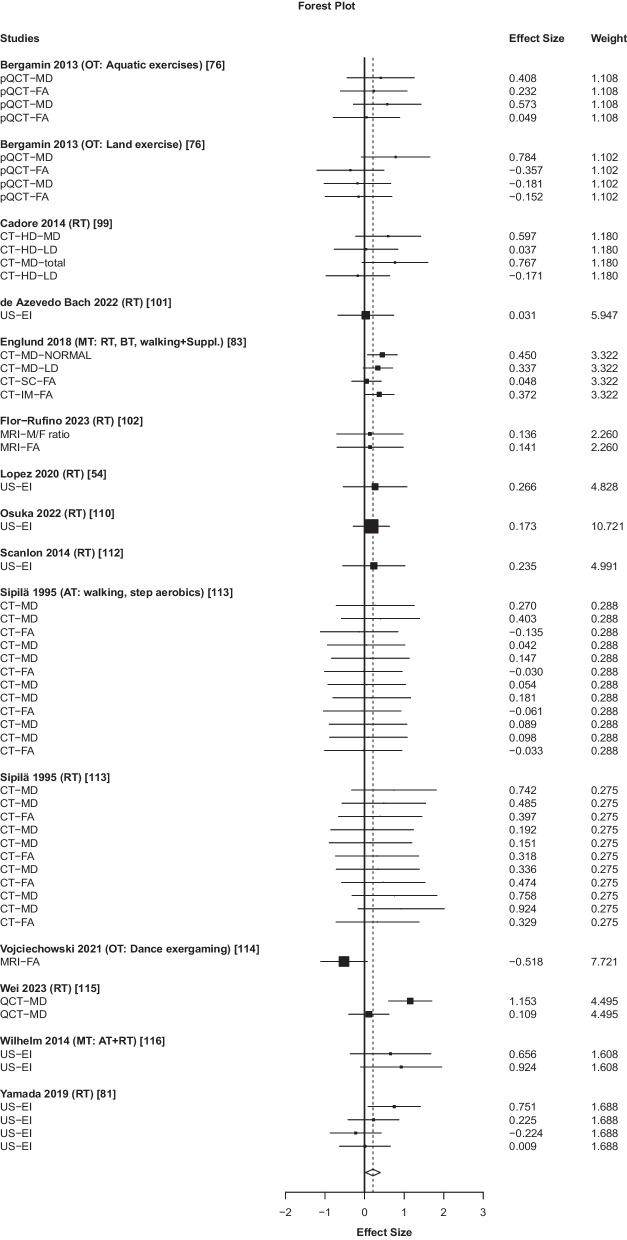
Effects of exercise interventions on morphological muscle quality in 562 participants, 13 studies, 15 intervention arms, and 54 outcomes (*g* = 0.21, 95% CI 0.03–0.40, *p* = 0.029, median *I*^2^ = 34%). Positive effect sizes denote favorable effects of exercise on morphological muscle quality. Abbreviations: AT, aerobic training; BT, balance training; CT, computed tomography; EI, echo intensity; FA, fat area; FA, fat density; HD, high density; IM, intramuscular; LD, low density; M/F, muscle to fat ratio; MD, muscle density; MRI, magnetic resonance imaging; MT, multimodal training; OT, other exercise training; pQCT, peripheral quantitative computed tomography; RT, resistance training; SC, subcutaneous; US, ultrasound imaging

While the overall and several subgroup analyses were statistically significant, most effect sizes were small, *g* ≤ 0.34, with the lower limits of the confidence intervals near zero, and study numbers as low as 4 (Table 2, Fig. 2). For example, the overall exercise-training effects on MMQ were *g* = 0.21 (small effect size, *n* = 562 participants, 95% CI 0.03–0.40, *p* = 0.029). When dietary supplementation was used, the effect of exercise on MMQ was *g* = 0.26. When supplementation was not used, it was *g* = 0.19. The highest effect size of *g* = 0.49 (medium effect size, *n* = 303 participants, 95% CI 0.12–0.86, *p* = 0.019) appeared for studies (*n* = 6) analyzing muscle density. Heterogeneity was low (median *I*^2^ = 16%).

**Table 2:** Overall and subgroup analyses for MMQ (upper panel) and NMQ (lower panel)

Variable	Effect size		95%CI			Heterogeneity		Frequencies			
	*g*	SE	LL	UL	*p*	*I*^2^	Tau^2^	*N*_*i*_	*k*	*N*_*s*_	*N*_*p*_
MMQ	0.21	0.08	0.03	0.40	0.029	25	0.04	15	54	13	562
*Population*											
LFOI	0.24	0.04	0.09	0.38	0.016	0	0.00	5	15	5	274
*Intervention*											
RT	0.28	0.08	0.08	0.47	0.013	8	0.01	9	27	9	319
OEI	0.12	0.18	− 0.37	0.62	0.538	45	0.11	6	27	5	243
*Intensity*											
Low	0.19	0.10	− 0.03	0.42	0.085	37	0.06	11	42	10	473
*Dietary supplementation*											
Yes	0.26	0.06	0.02	0.49	0.041	0	0.00	4	8	4	259
No	0.19	0.12	− 0.08	0.46	0.148	36	0.09	12	46	10	345
*Limb*											
Lower	0.16	0.08	− 0.03	0.35	0.088	0	0.00	14	48	12	502
*Method*											
CT	0.34	0.08	0.12	0.55	0.011	31	0.06	7	41	5	281
US	0.22	0.06	0.05	0.38	0.021	0	0.00	6	10	6	213
*Measure*											
EI	0.22	0.06	0.05	0.38	0.021	0	0.00	6	10	6	213
MD	0.49	0.15	0.12	0.86	0.019	29	0.05	8	28	6	303
*Control group*											
Passive	0.19	0.11	− 0.06	0.43	0.119	34	0.07	12	45	10	402
NMQ	0.68	0.16	0.35	1.01	0.000	81	0.6	26	55	21	894
*Population*											
HFOI	0.72	0.16	0.38	1.06	0.000	77	0.6	22	49	17	623
LFOI	0.51	0.52	− 1.15	2.16	0.400	91	0.7	4	6	4	271
*Intervention*											
RT	0.91	0.23	0.42	1.41	0.001	86	0.9	17	41	15	566
OEI	0.21	0.17 − 0.23 0.65 0.285	6	0.0	9	14	6	328			
*Intensity*											
Low	0.65	0.17	0.30	0.99	0.001	81	0.6	23	51	18	798
*Dietary supplementation*											
Yes	0.32	0.27	− 0.44	1.07	0.302	74	0.3	5	10	5	263
No	0.77	0.18	0.40	1.15	0.001	79	0.6	22	45	19	653
*Limb*											
Upper	0.61	0.28	− 0.02	1.24	0.056	76	0.6	11	20	8	302
Lower	0.74	0.19	0.35	1.13	0.001	83	0.7	24	33	19	844
*Muscle*											
Arm	0.61	0.28	− 0.02	1.24	0.056	76	0.6	11	20	8	302
Thigh	0.79	0.19	0.40	1.19	0.001	83	0.7	23	31	18	820
*Method*											
DXA	1.12	0.22	0.65	1.59	0.000	78	0.7	16	34	13	443
US	− 0.25	0.15	− 0.79	0.29	0.213	20	0.0	4	5	4	153
*Control group*											
Active	0.34	0.22	− 0.20	0.88	0.171	72	0.3	7	20	6	290
Passive	0.82	0.20	0.39	1.24	0.001	82	0.7	19	35	15	604

*g*, effect size; *SE*, standard error of the effect size; *CI, LL, UL*, 95% confidence interval lower and upper limit; *p*, probability; *I*^2^, percent of heterogeneity; *Tau*^2^, absolute value of true heterogeneity; *N*_*i*_, number of intervention arms; *k*, number of outcomes; *N*_*s*_, number of studies; *N*_*p*_, number of participants; *HFOI*, higher-functioning older individuals; *LFOI*, lower-functioning older individuals; *RT*, resistance training; *OEI*, other exercise interventions; *CT*, computed tomography; *US*, ultrasound imaging; *EI*, echo intensity; *MD*, muscle density

### Overall Effects and Subgroup Analyses of Exercise Effects on NMQ

**Fig. 3:**
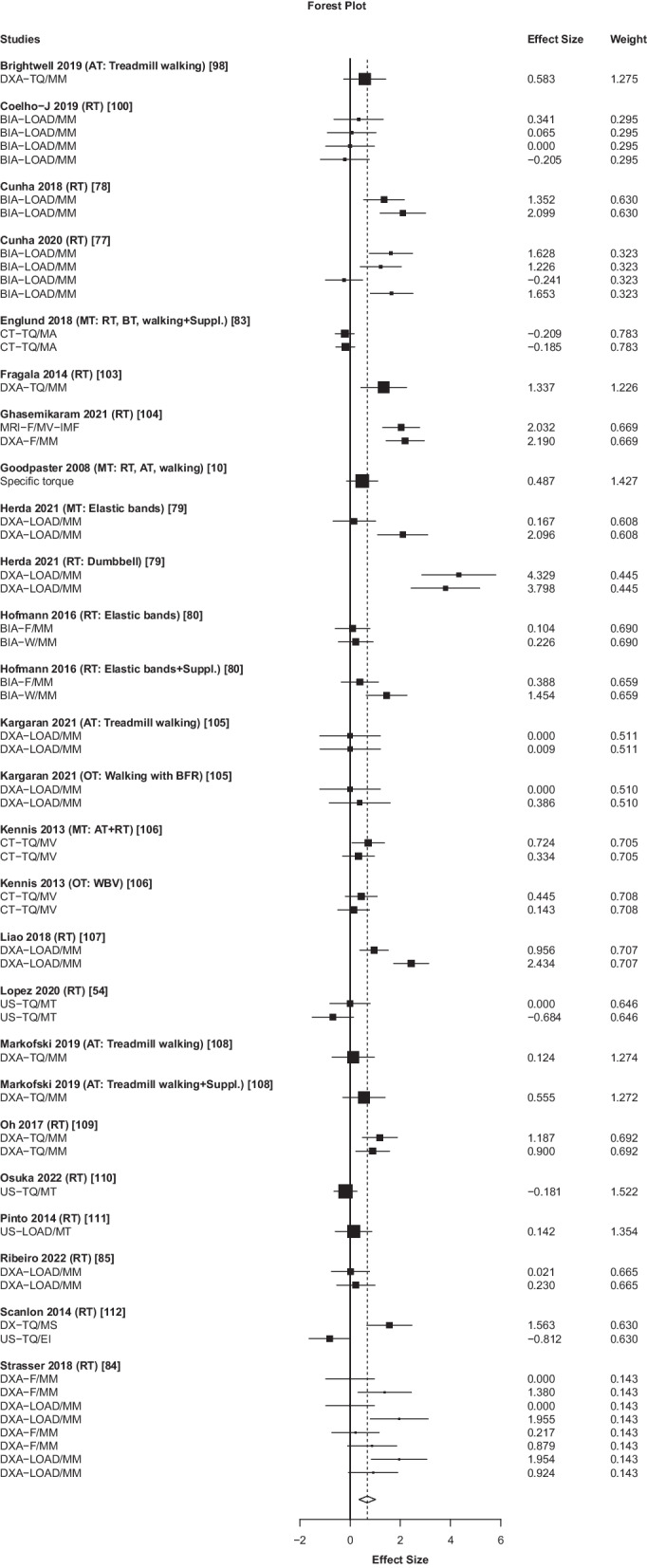
Effects of exercise interventions on neuromuscular muscle quality in 894 participants, 21 studies, 26 intervention arms, and 55 outcomes (*g* = 0.68, 95% CI 0.35–1.01, *p* < 0.001, median *I*^2^ = 79%). Positive effect sizes denote favorable effects of exercise on neuromuscular muscle quality. Abbreviations: AT, aerobic training; BFR, blood flow restriction; BIA, Bioelectrical impedance analysis; BT, balance training; CT, computed tomography; DXA, dual-energy X-ray absorptiometry; F, Force; MA, muscle area; MM, muscle mass; MRI, magnetic resonance imaging; MT, multimodal training; MT, muscle thickness; MV, muscle volume; OT, other exercise training; RT, resistance training; TQ, torque; W, weight

Exercise training had a medium overall effect on NMQ, *g* = 0.68 (95% CI 0.35–1.01, *p* < 0.001, *n* = 894 participants; Table 2, Fig. 3). Higher-functioning older adults responded strongly to the exercise training-stimulus (*g* = 0.72). Resistance training (*g* = 0.91) but no other exercise interventions (*g* = 0.21) improved NMQ. Low-intensity exercise improved NMQ (*g* = 0.65). When dietary supplementation was used, the effects of exercise on MMQ was *g* = 0.32. When supplementation was not used, it was *g* = 0.77. Exercise training-effects on NMQ were significant in lower (*g* = 0.74) but not in upper extremity muscles. Effect sizes were over two-fold greater when contrasting intervention effects with passive (*g* = 0.82) versus active (*g* = 0.34, n.s.) control groups. Data were insufficient to determine the exercise-training effects on NMQ in lower-functioning older adults (*n* = 4 studies), after high-intensity exercise, and methods to determine NMQ other than DXA. Heterogeneity was very high (median *I*^2^ = 79%).

### Effect Moderators

None of the variables moderated the effects of exercise on MMQ.

Additional file 1: Table S2 shows the final multi-variable meta-regression model for NMQ including training modality, intensity, and methods to assess NMQ. For training modality, the effects of resistance training versus other exercise interventions were greater (Δ*g* = 1.19, SE = 0.23, *p* < 0.001). The effects of high versus low intensity training were greater (Δ*g* = 0.57, SE = 0.13, *p* = 0.023) but the low number of high-intensity studies (*n* = 3) and resulting low degrees of freedom (< 4) make this moderating effect unreliable. Likewise, the moderating effects of methods to assess NMQ, although significant (*p* < 0.001), were considered unreliable because of the large imbalance in study numbers between methods (BIA: *n* = 2 studies; CT: *n* = 3; DXA: *n* = 13; MRI: *n* = 1; US: *n* = 4).

### Association Between MQ and Functional Outcomes

The associations between effect sizes reflecting changes in MMQ and effect sizes reflecting changes in functional outcomes as a whole (*n* = 13 studies, *p* = 0.873), mobility (*n* = 8, *p* = 0.888), and strength (*n* = 13, *p* = 0.588) were not significant. The associations between effect sizes reflecting changes in NMQ and effect sizes reflecting changes in mobility (*n* = 11 studies, *p* = 0.464) and balance (*n* = 8, *p* = 0.975) were not significant.

### Risk of Bias, Publication Bias, and Sensitivity Analyses

Additional file 1: Table S3 shows the risk of bias analysis. Of the 30 studies reporting on MQ, 1, 21, and 8 studies showed low, some or high concerns, respectively, with respect to overall bias.

The visual inspection of the funnel plots (Additional file 1: Figs. S1, S2) complemented with Egger’s regression tests for funnel plot asymmetry indicated potential publication bias resulting in possible overestimation of the summary effect on NMQ (*z* = 3.1, *p* = 0.002) but not on MMQ (*z* = 0.07, *p* = 0.947).

The sensitivity analyses exploring the effect of influential cases revealed that the removal of the identified influential cases (Additional file 1: Figs. S3, S4) did not affect the results of either MMQ (Δ*g* = − 0.086, SE = 0.147, *p* = 0.556) or NMQ analysis (Δ*g* = − 0.004, SE = 0.224, *p* = 0.984).

## Discussion

The purpose of this systematic scoping review with meta-analyses was to determine the effects and potential moderators of exercise training on MMQ and NMQ in lower- and higher-functioning healthy older individuals. In addition and in the form of a scoping review, we examined for the first time the effects of exercise training on MMQ and NMQ in individuals with neurological conditions. We found a medium-to-large effect [98] of exercise (e.g., resistance, aerobic, multimodal) training on healthy older individuals’ NMQ but small to no effects on MMQ. Multi-variable meta-regression revealed that resistance training, high intensity exercise, and assessment method of NMQ moderated the exercise effects on NMQ. However, the moderating effects of high intensity and assessment methods were likely unreliable due to the low number and average quality of included studies. We found no associations between exercise training-induced improvements in MQ outcomes and improvements in functional outcomes (muscle strength, power, mobility, balance). We did not identify any studies reporting exercise intervention effects on MQ in individuals with neurological conditions.

### Exercise Training Minimally Improves MMQ

Exercise interventions had small effects on MMQ (Fig. 2, Table 2). While a given exercise intervention could improve a given MMQ outcome with an effect size of 0.92 [116, 119], the aggregated effects of exercise on MMQ were comparable to the effects observed in passive control groups. Subgroup analyses of two populations, two categories of interventions, exercise training variables, muscle groups, dietary supplements, and assessment methods of MMQ revealed inconsistencies, negating the possibility of identifying potential moderators of exercise response on MMQ. Because between-study heterogeneity was low, the often-parallel changes in MMQ in the experimental and control groups after interventions imply the unreliability of the MMQ measures. Several studies reported *increases* in measures of myosteatosis of up to 9% following exercise training [76], or had 2-fold baseline between-group differences (exercise: 1,387 ± 723, control: 685 ± 146 cm^2^ low density [high fat infiltration] adipose tissue) [102] or reported other such unexpected findings. The small and inconsistent exercise-effects were additionally accompanied by a relatively high risk of bias (Additional file 1: Table S3). While we used a different mathematical treatment of the MMQ data to increase robustness of a potential aggregated effect, the current results crudely agree with a previous review reporting small effects of exercise training on MMQ in older adults [28]. We intended to extend these previous findings by determining the potential moderators and functional correlates of exercise training-induced changes in MMQ in individuals with and without neurological conditions. We argued that as the degree of sarcopenia and dynapenia increases with disease burden, as observed in individuals with neurological conditions and frailty [10, 72, 102, 120], the exercise training-effects on MMQ would be greater.

The small of effects of exercise training on MMQ do not diminish the physiological and functional significance of MMQ. In lower extremity muscles of older individuals and those with neurological conditions, adipose tissue accumulates between muscle fibers, between muscle groups, in the cytoplasm of muscle cells, and in the extramyocellular space in the form of interstitial or intramyofascial adipocytes. Such changes are accompanied by an activation of catabolic molecular cascades (i.e., Sirtuin1 gene activation) and ~ 40% increase in myostatin mRNA levels, counteracting myocellular growth. Ultimately, these effects result in insulin resistance, hyperinsulinemia, and cellular inflammation [44, 45, 121–123]. The small number of studies and relatively high risk of bias also prevented us from detecting systematic effects of the muscles trained (upper vs. lower extremity) and dietary supplements on MMQ. Therefore, the meta-analytical evidence from a past [28] and the current review calls for a renewed effort to determine the viability of MMQ to detect improvements in myosteatosis following chronic exercise training in lower and higher functioning older individuals and those with neurological conditions.

### Exercise Training Improves NMQ

Compared with MMQ, we found strikingly different results concerning the exercise training-effects on NMQ in healthy older individuals (Fig. 3, Table 2). While ratio scores can be statistically problematic, [124], muscle strength-to-muscle size ratio scores versus muscle strength or size alone allow for a more comprehensive estimation of exercise training-induced changes in muscle function [47, 48]. The low study numbers in lower-functioning older individuals (Additional file 1: Table S1) [10, 81, 102, 107] and in those with a neurological condition (*n* = 0 studies, see below) restricted our analyses to higher-functioning older individuals in whom our results were in line with a previous meta-synthesis of exercise-training effects on NMQ also in older individuals [28]. Our larger effect size suggests that resistance training (median duration: 12 weeks; frequency: 2×/week) is particularly effective in improving NMQ outcomes compared with other exercise interventions, including walking, running, aquatic, and dance exercise training (Table 2, Additional file 1: Table S1). Relative to all other types of exercise training, the multi-variable meta-regression confirmed that resistance training moderates the exercise effects on NMQ (Additional file 1: Table S2). While recent trends promote high-intensity exercise in older individuals regardless of health status [76, 107, 114, 119, 125], our data were inconclusive regarding exercise training-intensity effects on NMQ due to the low number of studies involving high-intensity exercise training (*n* = 3). On the other hand, it is encouraging that even low-intensity exercise training could improve NMQ in older individuals. This observation is in line with the specificity of exercise training with respect to baseline fitness (i.e., untrained individuals respond well to low-intensity exercise stimuli) [126]. Subgroup analyses also revealed actually lower effects of exercise with dietary supplements on NMQ compared with no-supplement training, a finding corroborated by another review of 22 studies [127]. It is possible that older individuals without nutrient deficiencies on a healthy diet would not benefit from dietary supplements, especially when receiving a strong anabolic stimulus from resistance training. The consistently greater effect sizes for muscle size outcomes in lower versus upper extremity muscles agree with most [74, 75, 128–130] but not all meta-analytical data [52]. Recorded for 10 h on each of two days, electromyogram activity of hand and arm muscles were active for 18% of the recording time [131]. Leg muscles were active for only 10% of the recording time. Upper-limb muscles were activated 67% more often than lower-limb muscles. However, when lower-limb muscles were activated, the mean amplitude of each burst was greater in leg muscles (~ 18% of maximum) compared with upper limb muscles (~ 7%). The greater effect sizes in muscle size outcomes in leg versus arm muscles could be because leg muscles are recruited and stimulated during the day more strongly than arm muscles. This greater activation along with exercise training aids the increases in muscle gains. That arm versus leg muscles are less intensely active might increase the need for higher training stimulus for hypertrophy.

Concerning the methods of measuring NMQ, there was a paucity of data (Table 2) and we can only confirm the DXA-based evaluation of the exercise-effects in older individuals (Table 2). However, the moderating effects of DXA versus other methods to determine NMQ, remain unclear due to low study numbers. As observed for MMQ, the functional relevance of these NMQ data remains unclear: there was no association between effect sizes reflecting exercise training-induced improvements in NMQ and effect sizes reflecting changes in muscle strength mobility and balance. The interpretation of our NMQ data requires further caution due to the high between-study heterogeneity, average study quality, and a potential risk of publication bias reflected by the asymmetrical funnel plot (Additional file 1: Fig. S2).

### Muscle Quality in Individuals with a Neurological Condition

We argued that if the degree of sarcopenia and dynapenia increased with the diagnosed disease burden, exercise effects on MQ would also increase with disease burden. Considering the potential physiological and functional significance of MQ in individuals with a neurological condition, it was unexpected that we would fail to identify a single study meeting our inclusion criteria. We thus provide a brief perspective on this issue.

Parkinson’s disease comprises bradykinetic movement disorders caused by dysfunctional basal ganglia-cortical neuronal circuits. Rigidity, postural instability, and gait dysfunction can co-occur with movement slowing. Clearly, MQ plays a key role in mobility in Parkinson’s disease. While the prevalence of dynapenia is more consistent and can approach 80%, the prevalence of sarcopenia varies widely between 6 [56] and 40% [58]. This large variation in sarcopenia stems from the interactions between aging, the disease, drugs, and physical inactivity [72, 125]. Sporadic data suggest that older individuals with Parkinson’s disease can have even higher fat-free mass than age-matched controls [132]. Physically active individuals with Parkinson’s disease compared with healthy individuals can also have similar maximal quadriceps force, volume, physiological cross-sectional area, and NMQ [133]. Individuals with this condition respond favorably to exercise training, implying improvements in muscle structure and function [56–58, 72, 120, 133–135]. However, studies are urgently needed to determine the effects and dose-response relations of exercise training on MQ in individuals with Parkinson’s disease.

Multiple sclerosis is a chronic autoimmune, inflammatory, demyelinating disease of the central nervous system causing poor motor-cognitive function and quality of life [136]. In addition to neuronal dysfunctions, impaired skeletal muscle metabolism, altered muscle fiber type composition, architecture, and impaired cross-bridge mechanics have been suggested to contribute to a disuse phenotype, overt fatigue, and muscle weakness in individuals with multiple sclerosis [137–140]. However, declines in muscle strength and muscle mass follow very different trajectories in these individuals, justifying the need to determine muscle function more accurately and reliably through MQ outcomes. Such measures would provide clearer insights into skeletal muscle functionality and adaptability following exercise training [60]. A determination of MMQ, including intramuscular non-contractile quantity in particular, after training is lacking [61, 63, 141]. Such data are especially needed to clarify the dissociation between improvements in muscle size and strength and functional outcomes [120, 141].

Stroke is the second leading cause of death worldwide. Nearly 50% of survivors live with chronic disability stemming from weakness, hypotrophy, fatigability, and altered motor control due to denervation, disuse, remodeling, and spasticity [68, 72]. Stroke-induced sarcopenia secondary to the cerebral trauma arises from immobilization and dysfunctional atrophy, impaired feeding, inflammation, sympathetic overactivation, and denervation. Unsurprisingly, the prevalence of sarcopenia in the acute stage of stroke is ~ 50% which improves to ~ 30% six months later [72, 121]. Skeletal muscle after a stroke is characterized by a ~ 25% increase in echo intensity, potentially indicative of high volumes of intramuscular fat deposition particularly in the paretic limb. Such changes are coupled with ~ 20% reductions in muscle mass and fiber size over 3 years [65, 72]. High-intensity resistance training, including eccentric overloading, has been effective in increasing muscle size, muscle strength (*g* = 0.59), walking capacity (*g* = 0.45) [64, 71, 142]. However, training studies so far have failed to capture the functionality of muscle adaptations by measuring intramuscular fat, a particularly strongly affected property of skeletal muscle in individuals with stroke.

In sum, considering the importance of skeletal muscle in metabolic, motor, and cognitive health, our review unexpectedly identified a substantial gap in our understanding of how exercise training modifies MQ in individuals with neurological conditions. We conjecture that quantifying responses to exercise interventions through MMQ and NMQ would benefit individuals with neurological conditions and therapists because such outcomes would permit: 1) direct, standardized comparisons of exercise adaptations in the contractile and non-contractile compartments of skeletal muscle with disease-free controls; 2) a more accurate determination of the functionality of newly acquired skeletal muscle mass, and 3) a more informed monitoring of disease progression, symptom management, and responses to drugs interacting with skeletal muscle metabolism and regulation.

### Limitations

The accuracy of stratifying older individuals into lower- and higher-functioning categories was limited by the number and type of tests provided in the individual studies and might not accurately reflect these health states. While exercise intensity is an important determinant of responses to training, our training intensity categorization was limited by the quality and quantity of information reported by the individual studies. The included studies were of average quality at best, mostly due to the process of randomization and measurement of the outcomes. For example, periodization of resistance training is an important training variable but due to a lack of details and insufficient data, we were unable to test its moderating effects on MQ. Due to the limited number of studies, the current review cannot inform exercise prescription concerning the most effective exercise interventions to improve MMQ and NMQ. Even for subgroup analyses with medium–high effect sizes, the median between-study heterogeneity was ~ 80% for NMQ outcomes, warranting serious caution in interpreting the data. A major limitation was a lack of studies in individuals with neurological conditions.

## Conclusion

Exercise training has small effects on MMQ and medium-large effects on NMQ in healthy older individuals. There was no association between improvements in MQ and increases in muscle strength, mobility, and balance. Information on dose-response relations following training is currently lacking. There is a critical gap in muscle quality data for older individuals with lower function and neurological conditions after exercise training. Well-designed studies are needed to examine the relevance of exercise training-induced changes in MQ to daily function in older individuals, especially in those with lower function and neurological conditions.

## Supplementary Information

**Additional file 1**. 1. Systematic search, syntaxes. 2. Supplementary Figures 1–5.3. Supplementary Tables 1–2.

## Data Availability

The R code used to analyze the data is freely available in the public domain under the links specified in the Statistical Analyses section.

## Abbreviations

IQR: Interquartile range
MQ: Muscle quality
MMQ: Morphological muscle quality
NMQ: Neuromuscular
MRI: Magnetic resonance imaging
pQCT: Peripheral quantitative computed tomography
CT: Computed tomography
US: Ultrasound imaging and also by
DXA: Dual-energy X-ray absorptiometry

## Declarations

### ChatGPT

All authors declare that they have not used ChatGPT or any other AI based tool for the writing of the manuscript.

## References

[1] Cruz-Jentoft AJ, Bahat G, Bauer J, Boirie Y, Bruyere O, Cederholm T (2019). Sarcopenia: revised European consensus on definition and diagnosis. Age Ageing.

[2] Frontera WR, Hughes VA, Fielding RA, Fiatarone MA, Evans WJ, Roubenoff R (2000). Aging of skeletal muscle: a 12-yr longitudinal study. J Appl Physiol (1985).

[3] Wolfe RR (2006). The underappreciated role of muscle in health and disease. Am J Clin Nutr.

[4] Faulkner JA, Larkin LM, Claflin DR, Brooks SV (2007). Age-related changes in the structure and function of skeletal muscles. Clin Exp Pharmacol Physiol.

[5] Deschenes MR (2004). Effects of aging on muscle fibre type and size. Sports Med.

[6] Suetta C, Haddock B, Alcazar J, Noerst T, Hansen OM, Ludvig H (2019). The Copenhagen Sarcopenia Study: lean mass, strength, power, and physical function in a Danish cohort aged 20–93 years. J Cachexia Sarcopenia Muscle.

[7] Janssen I, Heymsfield SB, Wang ZM, Ross R (2000). Skeletal muscle mass and distribution in 468 men and women aged 18–88 yr. J Appl Physiol (1985).

[8] Kennis E, Verschueren S, Van Roie E, Thomis M, Lefevre J, Delecluse C (2014). Longitudinal impact of aging on muscle quality in middle-aged men. Age (Dordr).

[9] Abe T, Thiebaud RS, Loenneke JP (2016). Age-related change in handgrip strength in men and women: is muscle quality a contributing factor?. Age (Dordr).

[10] Goodpaster BH, Chomentowski P, Ward BK, Rossi A, Glynn NW, Delmonico MJ (2008). Effects of physical activity on strength and skeletal muscle fat infiltration in older adults: a randomized controlled trial. J Appl Physiol (1985).

[11] Granacher U, Gruber M, Gollhofer A (2010). Force production capacity and functional reflex activity in young and elderly men. Aging Clin Exp Res.

[12] Manini TM, Clark BC (2012). Dynapenia and aging: an update. J Gerontol A Biol Sci Med Sci.

[13] Ferrucci L, de Cabo R, Knuth ND, Studenski S (2012). Of Greek heroes, wiggling worms, mighty mice, and old body builders. J Gerontol A Biol Sci Med Sci.

[14] Zanker J, Blackwell T, Patel S, Duchowny K, Brennan-Olsen S, Cummings SR (2022). Factor analysis to determine relative contributions of strength, physical performance, body composition and muscle mass to disability and mobility disability outcomes in older men. Exp Gerontol.

[15] Custodio Martins P, de Lima TR, Silva AM, Santos Silva DA (2022). Association of phase angle with muscle strength and aerobic fitness in different populations: a systematic review. Nutrition.

[16] Kato T, Ikezoe T, Tabara Y, Matsuda F, Tsuboyama T, Ichihashi N (2022). Differences in lower limb muscle strength and balance ability between sarcopenia stages depend on sex in community-dwelling older adults. Aging Clin Exp Res.

[17] Son JS, Chae SA, Testroet ED, Du M, Jun HP (2018). Exercise-induced myokines: a brief review of controversial issues of this decade. Expert Rev Endocrinol Metab.

[18] Yu JH, Kim REY, Jung JM, Park SY, Lee DY, Cho HJ (2021). Sarcopenia is associated with decreased gray matter volume in the parietal lobe: a longitudinal cohort study. BMC Geriatr.

[19] Wang J, Cao L, Xu S (2020). Sarcopenia affects clinical efficacy of immune checkpoint inhibitors in non-small cell lung cancer patients: a systematic review and meta-analysis. Int Immunopharmacol.

[20] Manrique-Espinoza B, Salinas-Rodriguez A, Rosas-Carrasco O, Gutierrez-Robledo LM, Avila-Funes JA (2017). Sarcopenia is associated with physical and mental components of health-related quality of life in older adults. J Am Med Dir Assoc.

[21] Verlaan S, Aspray TJ, Bauer JM, Cederholm T, Hemsworth J, Hill TR (2017). Nutritional status, body composition, and quality of life in community-dwelling sarcopenic and non-sarcopenic older adults: a case-control study. Clin Nutr.

[22] Oztorun HS, Bahsi R, Turgut T, Surmeli DM, Cosarderelioglu C, Atmis V (2022). The relationship between sarcopenia and central hemodynamics in older adults with falls: a cross-sectional study. Blood Press Monit.

[23] Rodrigues F, Domingos C, Monteiro D, Morouco P (2022). A review on aging, sarcopenia, falls, and resistance training in community-dwelling older adults. Int J Environ Res Public Health.

[24] Lim SK, Kong S (2022). Prevalence, physical characteristics, and fall risk in older adults with and without possible sarcopenia. Aging Clin Exp Res.

[25] Köller M. Sarcopenia-a geriatric pandemic: a narrative review. Wien Med Wochenschr. 2022. 10.1007/s10354-022-00927-0 35416610

[26] Xu J, Wan CS, Ktoris K, Reijnierse EM, Maier AB (2022). Sarcopenia is associated with mortality in adults: a systematic review and meta-analysis. Gerontology.

[27] Lopez P, Radaelli R, Taaffe DR, Newton RU, Galvao DA, Trajano GS (2021). Resistance training load effects on muscle hypertrophy and strength gain: systematic review and network meta-analysis. Med Sci Sports Exerc.

[28] Radaelli R, Taaffe DR, Newton RU, Galvao DA, Lopez P (2021). Exercise effects on muscle quality in older adults: a systematic review and meta-analysis. Sci Rep.

[29] Zhang H, Lin S, Gao T, Zhong F, Cai J, Sun Y (2018). Association between sarcopenia and metabolic syndrome in middle-aged and older non-obese adults: a systematic review and meta-analysis. Nutrients.

[30] Gao Q, Hu K, Gao J, Shang Y, Mei F, Zhao L (2022). Prevalence and prognostic value of sarcopenic obesity in patients with cancer: a systematic review and meta-analysis. Nutrition.

[31] Jogiat UM, Sasewich H, Turner SR, Baracos V, Eurich DT, Filafilo H (2022). Sarcopenia determined by skeletal muscle index predicts overall survival, disease-free survival, and postoperative complications in resectable esophageal cancer: a systematic review and meta-analysis. Ann Surg.

[32] Meyer HJ, Strobel A, Wienke A, Surov A. Prognostic role of low-skeletal muscle mass on staging computed tomography in metastasized colorectal cancer: a systematic review and meta-analysis. Clin Colorectal Cancer. 2022. 10.1016/j.clcc.2022.03.005 35792019

[33] Heo JE, Shim JS, Song BM, Bae HY, Lee HJ, Lee E (2018). Association between appendicular skeletal muscle mass and depressive symptoms: review of the cardiovascular and metabolic diseases etiology research center cohort. J Affect Disord.

[34] Salinas-Rodriguez A, Palazuelos-Gonzalez R, Rivera-Almaraz A, Manrique-Espinoza B (2021). Longitudinal association of sarcopenia and mild cognitive impairment among older Mexican adults. J Cachexia Sarcopenia Muscle.

[35] Orwoll ES, Blackwell T, Cummings SR, Cauley JA, Lane NE, Hoffman AR (2022). CT muscle density, D3Cr muscle mass, and body fat associations with physical performance, mobility outcomes, and mortality risk in older Men. J Gerontol A Biol Sci Med Sci.

[36] Smith C, Woessner MN, Sim M, Levinger I (2022). Sarcopenia definition: does it really matter? Implications for resistance training. Ageing Res Rev.

[37] Arango-Lopera VE, Arroyo P, Gutierrez-Robledo LM, Perez-Zepeda MU, Cesari M (2013). Mortality as an adverse outcome of sarcopenia. J Nutr Health Aging.

[38] Li CI, Liu CS, Lin CH, Yang SY, Li TC, Lin CC (2022). Independent and joint associations of skeletal muscle mass and physical performance with all-cause mortality among older adults: a 12-year prospective cohort study. BMC Geriatr.

[39] Lynch DH, Spangler HB, Franz JR, Krupenevich RL, Kim H, Nissman D (2022). Multimodal diagnostic approaches to advance precision medicine in sarcopenia and frailty. Nutrients.

[40] Kim JH, Cho JJ, Park YS (2015). Relationship between sarcopenic obesity and cardiovascular disease risk as estimated by the Framingham risk score. J Korean Med Sci.

[41] Longobucco Y, Krumpoch S, Lauretani F, Angileri V, Sieber C, Marzetti E (2022). Gait characteristics in community-dwelling older persons with low skeletal muscle mass and low physical performance. Aging Clin Exp Res.

[42] Gianoudis J, Bailey CA, Daly RM (2015). Associations between sedentary behaviour and body composition, muscle function and sarcopenia in community-dwelling older adults. Osteoporos Int.

[43] Cao L, Morley JE (2016). Sarcopenia is recognized as an independent condition by an international classification of disease, tenth revision, clinical modification (ICD-10-CM) code. J Am Med Dir Assoc.

[44] Fragala MS, Kenny AM, Kuchel GA (2015). Muscle quality in aging: a multi-dimensional approach to muscle functioning with applications for treatment. Sports Med.

[45] Correa-de-Araujo R, Addison O, Miljkovic I, Goodpaster BH, Bergman BC, Clark RV (2020). Myosteatosis in the context of skeletal muscle function deficit: an interdisciplinary workshop at the national institute on aging. Front Physiol.

[46] Barbat-Artigas S, Rolland Y, Zamboni M, Aubertin-Leheudre M (2012). How to assess functional status: a new muscle quality index. J Nutr Health Aging.

[47] Lynch NA, Metter EJ, Lindle RS, Fozard JL, Tobin JD, Roy TA (1999). Muscle quality. I. Age-associated differences between arm and leg muscle groups. J Appl Physiol (1985).

[48] Tracy BL, Ivey FM, Hurlbut D, Martel GF, Lemmer JT, Siegel EL (1999). Muscle quality. II. Effects of strength training in 65- to 75-yr-old men and women. J Appl Physiol (1985).

[49] Correa-de-Araujo R, Harris-Love MO, Miljkovic I, Fragala MS, Anthony BW, Manini TM (2017). The need for standardized assessment of muscle quality in skeletal muscle function deficit and other aging-related muscle dysfunctions: a symposium report. Front Physiol.

[50] Garatachea N, Pareja-Galeano H, Sanchis-Gomar F, Santos-Lozano A, Fiuza-Luces C, Moran M (2015). Exercise attenuates the major hallmarks of aging. Rejuvenation Res.

[51] Mende E, Moeinnia N, Schaller N, Weiss M, Haller B, Halle M (2022). Progressive machine-based resistance training for prevention and treatment of sarcopenia in the oldest old: a systematic review and meta-analysis. Exp Gerontol.

[52] Zhang Y, Zou L, Chen ST, Bae JH, Kim DY, Liu X (2021). Effects and moderators of exercise on sarcopenic components in sarcopenic elderly: a systematic review and meta-analysis. Front Med (Lausanne).

[53] Teodoro JL, Izquierdo M, da Silva LXN, Baroni BM, Grazioli R, Lopez P (2020). Effects of long-term concurrent training to failure or not in muscle power output, muscle quality and cardiometabolic risk factors in older men: a secondary analysis of a randomized clinical trial. Exp Gerontol.

[54] Lopez P, Crosby BJ, Robetti BP, Turella DJP, Weber TAS, de Oliveira ML (2020). Effects of an 8-week resistance training intervention on plantar flexor muscle quality and functional capacity in older women: a randomised controlled trial. Exp Gerontol.

[55] Radaelli R, Brusco CM, Lopez P, Rech A, Machado CLF, Grazioli R (2019). Muscle quality and functionality in older women improve similarly with muscle power training using one or three sets. Exp Gerontol.

[56] Barichella M, Pinelli G, Iorio L, Cassani E, Valentino A, Pusani C (2016). Sarcopenia and dynapenia in patients with parkinsonism. J Am Med Dir Assoc.

[57] Do Nascimento TG, Paes-Silva RP, Da Luz MCL, Cabral PC, De Araujo Bezerra GK, Gomes ACB (2022). Phase angle, muscle mass, and functionality in patients with Parkinson's disease. Neurol Sci.

[58] Vetrano DL, Pisciotta MS, Laudisio A, Lo Monaco MR, Onder G, Brandi V (2018). Sarcopenia in Parkinson disease: comparison of different criteria and association with disease severity. J Am Med Dir Assoc.

[59] Diechmann MD, Campbell E, Coulter E, Paul L, Dalgas U, Hvid LG (2021). Effects of exercise training on neurotrophic factors and subsequent neuroprotection in persons with multiple sclerosis-a systematic review and meta-analysis. Brain Sci.

[60] Portilla-Cueto K, Medina-Perez C, Romero-Perez EM, Nunez-Othon G, Horta-Gim MA, de Paz JA (2022). Muscle quality of knee extensors based on several types of force in multiple sclerosis patients with varying degrees of disability. Medicina (Kaunas).

[61] Wens I, Dalgas U, Vandenabeele F, Grevendonk L, Verboven K, Hansen D (2015). High intensity exercise in multiple sclerosis: effects on muscle contractile characteristics and exercise capacity, a randomised controlled trial. PLoS ONE.

[62] Dalgas U, Stenager E, Jakobsen J, Petersen T, Overgaard K, Ingemann-Hansen T (2010). Muscle fiber size increases following resistance training in multiple sclerosis. Mult Scler.

[63] Kjolhede T, Vissing K, de Place L, Pedersen BG, Ringgaard S, Stenager E (2015). Neuromuscular adaptations to long-term progressive resistance training translates to improved functional capacity for people with multiple sclerosis and is maintained at follow-up. Mult Scler.

[64] Fernandez-Gonzalo R, Fernandez-Gonzalo S, Turon M, Prieto C, Tesch PA, Garcia-Carreira MC (2016). Muscle, functional and cognitive adaptations after flywheel resistance training in stroke patients: a pilot randomized controlled trial. J Neuroeng Rehabil.

[65] Akazawa N, Harada K, Okawa N, Kishi M, Tamura K, Moriyama H (2021). Changes in quadriceps thickness and echo intensity in chronic stroke survivors: a 3-year longitudinal study. J Stroke Cerebrovasc Dis.

[66] Feng D, Zhao C, Chen D, He X, Lu X, Gao W (2021). Preventive effect of rehabilitation training therapy on muscle quality in patients with stroke. Retrosp Study Aging Pathobiol Ther.

[67] Honaga K, Mori N, Akimoto T, Tsujikawa M, Kawakami M, Okamoto T (2022). Investigation of the effect of nutritional supplementation with whey protein and vitamin D on muscle mass and muscle quality in subacute post-stroke rehabilitation patients: a randomized, single-blinded, placebo-controlled trial. Nutrients.

[68] Mas MF, Gonzalez J, Frontera WR (2020). Stroke and sarcopenia. Curr Phys Med Rehabil Rep.

[69] Ryan AS, Ivey FM, Prior S, Li G, Hafer-Macko C (2011). Skeletal muscle hypertrophy and muscle myostatin reduction after resistive training in stroke survivors. Stroke.

[70] Suzuki K, Ito T, Okada Y, Hiraoka T, Hanayama K, Tsubahara A (2020). Preventive effects of repetitive peripheral magnetic stimulation on muscle atrophy in the paretic lower limb of acute stroke patients: a pilot study. Prog Rehabil Med.

[71] Tanaka S, Ito D, Kimura Y, Ishiyama D, Suzuki M, Koyama S (2022). Relationship between longitudinal changes in skeletal muscle characteristics over time and functional recovery during intensive rehabilitation of patients with subacute stroke. Top Stroke Rehabil.

[72] Dalise S, Azzollini V, Chisari C (2020). Brain and muscle: how central nervous system disorders can modify the skeletal muscle. Diagnostics (Basel).

[73] Harbo T, Brincks J, Andersen H (2012). Maximal isokinetic and isometric muscle strength of major muscle groups related to age, body mass, height, and sex in 178 healthy subjects. Eur J Appl Physiol.

[74] Phillipe de Lucena Alves C, Camara M, Dantas Macedo GA, Freire YA, de Melo Silva R, Paulo-Pereira R, et al. Agreement between upper and lower limb measures to identify older adults with low skeletal muscle strength, muscle mass and muscle quality. PLoS ONE. 2022;17(1):e0262732. 10.1371/journal.pone.0262732 PMC8782376 35061817

[75] Candow DG, Chilibeck PD (2005). Differences in size, strength, and power of upper and lower body muscle groups in young and older men. J Gerontol A Biol Sci Med Sci.

[76] Bergamin M, Ermolao A, Tolomio S, Berton L, Sergi G, Zaccaria M (2013). Water- versus land-based exercise in elderly subjects: effects on physical performance and body composition. Clin Interv Aging.

[77] Cunha PM, Nunes JP, Tomeleri CM, Nascimento MA, Schoenfeld BJ, Antunes M (2020). Resistance training performed with single and multiple sets induces similar improvements in muscular strength, muscle mass, muscle quality, and IGF-1 in older women: a randomized controlled trial. J Strength Cond Res.

[78] Cunha PM, Tomeleri CM, Nascimento MAD, Nunes JP, Antunes M, Nabuco HCG (2018). Improvement of cellular health indicators and muscle quality in older women with different resistance training volumes. J Sports Sci.

[79] Herda AA, Nabavizadeh O (2021). Short-term resistance training in older adults improves muscle quality: a randomized control trial. Exp Gerontol.

[80] Hofmann M, Schober-Halper B, Oesen S, Franzke B, Tschan H, Bachl N (2016). Effects of elastic band resistance training and nutritional supplementation on muscle quality and circulating muscle growth and degradation factors of institutionalized elderly women: the Vienna Active Ageing Study (VAAS). Eur J Appl Physiol.

[81] Yamada M, Kimura Y, Ishiyama D, Nishio N, Otobe Y, Tanaka T (2019). Synergistic effect of bodyweight resistance exercise and protein supplementation on skeletal muscle in sarcopenic or dynapenic older adults. Geriatr Gerontol Int.

[82] Beaudart C, Dawson A, Shaw SC, Harvey NC, Kanis JA, Binkley N (2017). Nutrition and physical activity in the prevention and treatment of sarcopenia: systematic review. Osteoporos Int.

[83] Englund DA, Kirn DR, Koochek A, Zhu H, Travison TG, Reid KF (2017). Nutritional supplementation with physical activity improves muscle composition in mobility-limited older adults, the VIVE2 study: a randomized, double-blind, placebo-controlled trial. J Gerontol A Biol Sci Med Sci.

[84] Strasser EM, Hofmann M, Franzke B, Schober-Halper B, Oesen S, Jandrasits W (2018). Strength training increases skeletal muscle quality but not muscle mass in old institutionalized adults: a randomized, multi-arm parallel and controlled intervention study. Eur J Phys Rehabil Med.

[85] Ribeiro AS, Picoloto A, Nunes JP, Bezerra ES, Schoenfeld BJ, Cyrino ES (2022). Effects of different resistance training loads on the muscle quality index in older women. J Strength Cond Res.

[86] Middleton A, Fritz SL, Lusardi M (2015). Walking speed: the functional vital sign. J Aging Phys Act.

[87] Bernabei R, Landi F, Calvani R, Cesari M, Del Signore S, Anker SD (2022). Multicomponent intervention to prevent mobility disability in frail older adults: randomised controlled trial (SPRINTT project). BMJ.

[88] Kraemer WJ, Adams K, Cafarelli E, Dudley GA, Dooly C, Feigenbaum MS (2002). American College of Sports Medicine position stand. Progression models in resistance training for healthy adults. Med Sci Sports Exerc.

[89] Ratamess NA, Alvar BA, Evetoch TK, Housh TJ, Kibler WB, Kraemer WJ (2009). Progression models in resistance training for healthy adults. Med Sci Sports Exerc.

[90] Borde R, Hortobagyi T, Granacher U (2015). Dose-response relationships of resistance training in healthy old adults: a systematic review and meta-analysis. Sports Med.

[91] Ainsworth BE, Haskell WL, Whitt MC, Irwin ML, Swartz AM, Strath SJ (2000). Compendium of physical activities: an update of activity codes and MET intensities. Med Sci Sports Exerc.

[92] Garber CE, Blissmer B, Deschenes MR, Franklin BA, Lamonte MJ, Lee IM (2011). American College of Sports Medicine position stand. Quantity and quality of exercise for developing and maintaining cardiorespiratory, musculoskeletal, and neuromotor fitness in apparently healthy adults: guidance for prescribing exercise. Med Sci Sports Exerc.

[93] Sterne JAC, Savovic J, Page MJ, Elbers RG, Blencowe NS, Boutron I (2019). RoB 2: a revised tool for assessing risk of bias in randomised trials. BMJ.

[94] Hedges LV, Tipton E, Johnson MC (2010). Robust variance estimation in meta-regression with dependent effect size estimates. Res Synth Meth.

[95] Tipton E (2015). Small sample adjustments for robust variance estimation with meta-regression. Psych Meth.

[96] Viechtbauer W, Cheung MW (2010). Outlier and influence diagnostics for meta-analysis. Res Synth Methods.

[97] Sterne JA, Egger M, Rothstein HR, Sutton AJ, Borenstein M (2005). Regression methods to detect publication and other bias in meta-analysis. Publication bias in meta-analysis: prevention, assessment and adjustments.

[98] Brydges CR (2019). Effect size guidelines, sample size calculations, and statistical power in gerontology. Innov Aging.

[99] Higgins JP, Thompson SG, Deeks JJ, Altman DG (2003). Measuring inconsistency in meta-analyses. BMJ.

[100] Falck RS, Davis JC, Best JR, Crockett RA, Liu-Ambrose T (2019). Impact of exercise training on physical and cognitive function among older adults: a systematic review and meta-analysis. Neurobiol Aging.

[101] Brightwell CR, Markofski MM, Moro T, Fry CS, Porter C, Volpi E (2019). Moderate-intensity aerobic exercise improves skeletal muscle quality in older adults. Transl Sports Med.

[102] Cadore EL, Casas-Herrero A, Zambom-Ferraresi F, Idoate F, Millor N, Gomez M (2014). Multicomponent exercises including muscle power training enhance muscle mass, power output, and functional outcomes in institutionalized frail nonagenarians. Age (Dordr).

[103] Coelho-Junior HJ, de Oliveira GI, Sampaio RAC, Sewo Sampaio PY, Cadore EL, Izquierdo M (2019). Periodized and non-periodized resistance training programs on body composition and physical function of older women. Exp Gerontol.

[104] de Azevedo BS, Radaelli R, Beck Schemes M, Neske R, Garbelotto C, Roschel H (2022). Can supplemental protein to low-protein containing meals superimpose on resistance-training muscle adaptations in older adults? A randomized clinical trial. Exp Gerontol.

[105] Flor-Rufino C, Barrachina-Igual J, Perez-Ros P, Pablos-Monzo A, Sanz-Requena R, Martinez-Arnau FM (2023). Fat infiltration and muscle hydration improve after high-intensity resistance training in women with sarcopenia A randomized clinical trial. Maturitas.

[106] Fragala MS, Jajtner AR, Beyer KS, Townsend JR, Emerson NS, Scanlon TC (2014). Biomarkers of muscle quality: N-terminal propeptide of type III procollagen and C-terminal agrin fragment responses to resistance exercise training in older adults. J Cachexia Sarcopenia Muscle.

[107] Ghasemikaram M, Engelke K, Kohl M, von Stengel S, Kemmler W (2021). Detraining effects on muscle quality in older men with osteosarcopenia. Follow-up of the randomized controlled franconian osteopenia and sarcopenia trial (FrOST). Nutrients.

[108] Kargaran A, Abedinpour A, Saadatmehr Z, Yaali R, Amani-Shalamzari S, Gahreman D (2021). Effects of dual-task training with blood flow restriction on cognitive functions, muscle quality, and circulatory biomarkers in elderly women. Physiol Behav.

[109] Kennis E, Verschueren SM, Bogaerts A, Coudyzer W, Boonen S, Delecluse C (2013). Effects of fitness and vibration training on muscle quality: a 1-year postintervention follow-up in older men. Arch Phys Med Rehabil.

[110] Liao CD, Tsauo JY, Huang SW, Ku JW, Hsiao DJ, Liou TH (2018). Effects of elastic band exercise on lean mass and physical capacity in older women with sarcopenic obesity: a randomized controlled trial. Sci Rep.

[111] Markofski MM, Jennings K, Timmerman KL, Dickinson JM, Fry CS, Borack MS (2019). Effect of aerobic exercise training and essential amino acid supplementation for 24 weeks on physical function, body composition, and muscle metabolism in healthy, independent older adults: a randomized clinical trial. J Gerontol A Biol Sci Med Sci.

[112] Oh SL, Kim HJ, Woo S, Cho BL, Song M, Park YH (2017). Effects of an integrated health education and elastic band resistance training program on physical function and muscle strength in community-dwelling elderly women: healthy aging and happy aging II study. Geriatr Gerontol Int.

[113] Osuka Y, Kojima N, Nishihara K, Sasai H, Wakaba K, Tanaka K (2022). Beta-hydroxy-beta-methylbutyrate supplementation may not enhance additional effects of exercise on muscle quality in older women. Med Sci Sports Exerc.

[114] Pinto RS, Correa CS, Radaelli R, Cadore EL, Brown LE, Bottaro M (2014). Short-term strength training improves muscle quality and functional capacity of elderly women. Age (Dordr).

[115] Scanlon TC, Fragala MS, Stout JR, Emerson NS, Beyer KS, Oliveira LP (2014). Muscle architecture and strength: adaptations to short-term resistance training in older adults. Muscle Nerve.

[116] Sipila S, Suominen H (1995). Effects of strength and endurance training on thigh and leg muscle mass and composition in elderly women. J Appl Physiol (1985).

[117] Vojciechowski AS, Silva CTS, Rodrigues EV, Gallo LH, Melo Filho J, Gomes ARS (2021). Does physical dance training with virtual games change muscle quality of community-dwelling older women?. Games Health J.

[118] Wei M, Meng D, Guo H, He S, Tian Z, Wang Z (2022). Hybrid exercise program for sarcopenia in older adults: the effectiveness of explainable artificial intelligence-based clinical assistance in assessing skeletal muscle area. Int J Environ Res Public Health.

[119] Wilhelm EN, Rech A, Minozzo F, Botton CE, Radaelli R, Teixeira BC (2014). Concurrent strength and endurance training exercise sequence does not affect neuromuscular adaptations in older men. Exp Gerontol.

[120] Hortobágyi T, Granacher U, Fernandez-Del-Olmo M, Howatson G, Manca A, Deriu F (2021). Functional relevance of resistance training-induced neuroplasticity in health and disease. Neurosci Biobehav Rev.

[121] Azzollini V, Dalise S, Chisari C (2021). How does stroke affect skeletal muscle? State of the art and rehabilitation perspective. Front Neurol.

[122] Engelke K, Ghasemikaram M, Chaudry O, Uder M, Nagel AM, Jakob F (2022). The effect of ageing on fat infiltration of thigh and paraspinal muscles in men. Aging Clin Exp Res.

[123] Sardeli AV, Tomeleri CM, Cyrino ES, Fernhall B, Cavaglieri CR, Chacon-Mikahil MPT (2018). Effect of resistance training on inflammatory markers of older adults: a meta-analysis. Exp Gerontol.

[124] Curran-Everett D (2013). Explorations in statistics: the analysis of ratios and normalized data. Adv Physiol Educ.

[125] Dibble LE, Hale TF, Marcus RL, Droge J, Gerber JP, LaStayo PC (2006). High-intensity resistance training amplifies muscle hypertrophy and functional gains in persons with Parkinson's disease. Mov Disord.

[126] Brahms CM, Hortobágyi T, Kressig RW, Granacher U (2021). The interaction between mobility status and exercise specificity in older adults. Exerc Sport Sci Rev.

[127] Choi M, Kim H, Bae J (2021). Does the combination of resistance training and a nutritional intervention have a synergic effect on muscle mass, strength, and physical function in older adults? A systematic review and meta-analysis. BMC Geriatr.

[128] Antunes M, Kassiano W, Silva AM, Schoenfeld BJ, Ribeiro AS, Costa B (2022). Volume reduction: which dose is sufficient to retain resistance training adaptations in older women?. Int J Sports Med.

[129] Radaelli R, Botton CE, Wilhelm EN, Bottaro M, Brown LE, Lacerda F (2014). Time course of low- and high-volume strength training on neuromuscular adaptations and muscle quality in older women. Age (Dordr).

[130] Sousa N, Mendes R, Abrantes C, Sampaio J (2011). Differences in maximum upper and lower limb strength in older adults after a 12 week intense resistance training program. J Hum Kinet.

[131] Kern DS, Semmler JG, Enoka RM (2001). Long-term activity in upper- and lower-limb muscles of humans. J Appl Physiol (1985).

[132] Cereda E, Cassani E, Barichella M, Spadafranca A, Caccialanza R, Bertoli S (2012). Low cardiometabolic risk in Parkinson's disease is independent of nutritional status, body composition and fat distribution. Clin Nutr.

[133] Martignon C, Ruzzante F, Giuriato G, Laginestra FG, Pedrinolla A, Di Vico IA (2021). The key role of physical activity against the neuromuscular deterioration in patients with Parkinson's disease. Acta Physiol (Oxf).

[134] Kelly NA, Ford MP, Standaert DG, Watts RL, Bickel CS, Moellering DR (2014). Novel, high-intensity exercise prescription improves muscle mass, mitochondrial function, and physical capacity in individuals with Parkinson's disease. J Appl Physiol (1985).

[135] Chiang PL, Chen YS, Lin AW. Altered body composition of psoas and thigh muscles in relation to frailty and severity of Parkinson's Disease. Int J Environ Res Public Health. 2019;16(19). 10.3390/ijerph16193667 PMC6801975 31569569

[136] Reich DS, Lucchinetti CF, Calabresi PA (2018). Multiple sclerosis. N Engl J Med.

[137] Kirmaci ZIK, Firat T, Ozkur HA, Neyal AM, Neyal A, Ergun N. Muscle architecture and its relationship with lower extremity muscle strength in multiple sclerosis. Acta Neurol Belg. 2021. 10.1007/s13760-021-01768-1 34417688

[138] Carroll CC, Gallagher PM, Seidle ME, Trappe SW (2005). Skeletal muscle characteristics of people with multiple sclerosis. Arch Phys Med Rehabil.

[139] Kent-Braun JA, Ng AV, Castro M, Weiner MW, Gelinas D, Dudley GA (1997). Strength, skeletal muscle composition, and enzyme activity in multiple sclerosis. J Appl Physiol (1985).

[140] Ramsay JW, Barrance PJ, Buchanan TS, Higginson JS (2011). Paretic muscle atrophy and non-contractile tissue content in individual muscles of the post-stroke lower extremity. J Biomech.

[141] Taul-Madsen L, Connolly L, Dennett R, Freeman J, Dalgas U, Hvid LG (2021). Is aerobic or resistance training the most effective exercise modality for improving lower extremity physical function and perceived fatigue in people with multiple sclerosis? A systematic review and meta-analysis. Arch Phys Med Rehabil.

[142] Lee J, Stone AJ (2020). Combined aerobic and resistance training for cardiorespiratory fitness, muscle strength, and walking capacity after stroke: a systematic review and meta-analysis. J Stroke Cerebrovasc Dis.

