# Supplementary material for: Effects of Exercise Training on Muscle Quality in Older Individuals: A Systematic Scoping Review with Meta-Analyses
Source: Sports Med Open. 2023 Jun 6;9:41. doi: 10.1186/s40798-023-00585-5 (PMC10244313; doi:10.1186/s40798-023-00585-5)
Supplement: Supplementary file 1 — Additional file 1. 1. Systematic search, syntaxes. 2. Supplementary Figures 1–5.3. Supplementary Tables 1–2. [file 40798_2023_585_MOESM1_ESM.docx]

**ONLINE SUPPLEMENTARY MATERIALS**

1. Systematic search, syntaxes

2. Supplementary Figures 1-5

3. Supplementary Tables 1-2

**1. Systematic search, syntaxes**

We conducted computerized systematic literature searches in PubMed, which was adapted to Embase and Web of Science Core Collection (Clarivate Analytics) with a closing date of July 26, 2022. We followed PRISMA review guidelines ^1^. The syntax was formulated so that we could determine the effects of exercise training interventions on muscle quality and motor function outcomes in healthy in healthy and mobility-limited older adults with or without neurological disease. The three main syntax terms were: population, exercise intervention, and muscle quality linked by Boolean AND, OR operators. The review protocol is registered with the PROSPERO database (CRD42021285802).

**Reference**

1. Page MJ, McKenzie JE, Bossuyt PM, et al. The PRISMA 2020 statement: an updated guideline for reporting systematic reviews. *Rev Esp Cardiol (Engl Ed)* 2021;74(9):790-99. doi: 10.1016/j.rec.2021.07.010 [published Online First: 2021/08/28]

**Syntaxes**

**1. PubMed**

("Aged"[Mesh] OR "Geriatric Assessment"[Mesh] OR elderly[tiab] OR older-patient*[tiab] OR older-person*[tiab] OR older-people[tiab] OR older-men[tiab] OR older-women[tiab] OR older-subject*[tiab] OR older-adult*[tiab] OR old-patient*[tiab] OR old-person*[tiab] OR old-people[tiab] OR old-adult*[tiab] OR geriatr*[tiab] OR “Parkinson Disease”[Mesh] OR Parkinson diseas*[tiab] OR “stroke”[Mesh] OR stroke[tiab] OR “brain Ischemia”[tiab] OR “intracranial hemorrhages”[tiab] OR “Multiple Sclerosis”[Mesh] OR multiple sclerosis[tiab] OR “Sarcopenia”[Mesh] OR “Muscular Atrophy”[Mesh] OR sarcopenia*[tiab] OR “Hip Fractures”[Mesh] OR hip fractures[tiab] OR femoral neck fracture*[tiab])

AND

("Exercise Therapy"[Mesh] OR "Motor Activity"[Mesh] OR "Physical Fitness"[Mesh] OR strength-training[tiab] OR resistance-training[tiab] OR exercise-intervention*[tiab] OR exercise[ti] OR vibration-training[tiab] OR physical-exercise[tiab] OR physical-activity[tiab] OR aerobic-exercise[tiab])

AND

(“muscle quality” [tiab] OR “specific tension” [tiab] OR “echo intensity” [tiab] OR echogenicity [tiab] OR “intermuscular adipose tissue” [tiab] OR “intermuscular fat” [tiab] OR “intramuscular adipose tissue” [tiab] OR “intramuscular fat” [tiab] OR “muscle attenuation” [tiab] OR “muscle density” [tiab] OR “radiological density” [tiab] OR “muscle composition” [tiab] OR “muscle fat infiltration” [tiab] OR “myosteatosis” [tiab] OR muscle architecture[tiab] AND ("Muscle Strength"[Mesh] OR muscle-strength[tiab] OR muscular-strength[tiab] OR "Muscle, Skeletal"[Mesh] OR muscle*[tiab] OR functional-capacity[tiab] OR physical-function*[tiab] OR muscle-thickness[tiab] OR muscle-mass[tiab]))

AND

("Randomized Controlled Trial" [Publication Type] OR random*[tiab] OR randomized controlled trial[tiab] OR controlled-study[tiab] OR clinical-study[tiab] OR controlled-trial[tiab] OR trial[tiab] OR groups[tiab] OR controlled clinical trial[tiab])

**2. Embase**

(‘Aged’/exp OR ‘Geriatric Assessment‘/exp OR ‘Parkinson Disease‘/exp OR ‘cerebrovascular accident‘/exp OR ‘Multiple Sclerosis‘/exp OR ‘Sarcopenia‘/exp OR ‘Muscule Atrophy‘/exp OR ‘Hip Fracture‘/exp OR ‘elderly’:ti,ab,kw OR ‘older-patient*’:ti,ab,kw OR ‘older-person*’:ti,ab,kw OR ‘older-people’:ti,ab,kw OR ‘older-men’:ti,ab,kw OR ‘older-women’:ti,ab,kw OR ‘older-subject*’:ti,ab,kw OR ‘older-adult*’:ti,ab,kw OR ‘old-patient*’:ti,ab,kw OR ‘old-person*’:ti,ab,kw OR ‘old-people’:ti,ab,kw OR ‘old-adult*’:ti,ab,kw OR ‘geriatr*’:ti,ab,kw OR ‘Parkinson diseas*’:ti,ab,kw OR ‘stroke’:ti,ab,kw OR ‘brain Ischemia’:ti,ab,kw OR ‘intracranial hemorrhages’:ti,ab,kw OR ‘multiple sclerosis’:ti,ab,kw OR ‘sarcopenia*’:ti,ab,kw OR ‘hip fractures’:ti,ab,kw OR ‘femoral neck fracture*’:ti,ab,kw)

AND

(‘kinesiotherapy‘/exp OR ‘Motor Activity‘/exp OR ‘Fitness‘/exp OR ‘strength-training’:ti,ab,kw OR ‘resistance-training’:ti,ab,kw OR ‘exercise-intervention*’:ti,ab,kw OR ‘exercise’:ti,ab,kw OR ‘vibration-training’:ti,ab,kw OR ‘physical-exercise’:ti,ab,kw OR ‘physical-activity’:ti,ab,kw OR ‘aerobic-exercise’:ti,ab,kw)

AND

(‘muscle quality’:ti,ab,kw OR ‘specific tension’:ti,ab,kw OR ‘echo intensity’:ti,ab,kw OR ‘echogenicity’:ti,ab,kw OR ‘intermuscular adipose tissue’:ti,ab,kw OR ‘intermuscular fat’:ti,ab,kw OR ‘intramuscular adipose tissue’:ti,ab,kw OR ‘intramuscular fat’:ti,ab,kw OR ‘muscle attenuation’:ti,ab,kw OR ‘muscle density’:ti,ab,kw OR ‘radiological density’:ti,ab,kw OR ‘muscle composition’:ti,ab,kw OR ‘muscle fat infiltration’:ti,ab,kw OR ‘myosteatosis’:ti,ab,kw OR ‘muscle architecture’:ti,ab,kw AND (Muscle Strength/exp OR skeletal muscle/exp OR ‘muscle-strength’:ti,ab,kw OR ‘muscular-strength’:ti,ab,kw OR ‘muscle*’:ti,ab,kw OR ‘functional-capacity’:ti,ab,kw OR ‘physical-function*’:ti,ab,kw OR ‘muscle-thickness’:ti,ab,kw OR ‘muscle-mass’:ti,ab,kw))

AND

(‘randomized controlled trial‘/exp OR ‘random*’:ti,ab,kw OR ‘randomized controlled trial’:ti,ab,kw OR ‘controlled-study’:ti,ab,kw OR ‘clinical-study’:ti,ab,kw OR ‘controlled-trial’:ti,ab,kw OR ‘trial’:ti,ab,kw OR ‘groups’:ti,ab,kw OR ‘controlled clinical trial’:ti,ab,kw)

**3. Web of Science**

TI=("Aged" OR "Geriatric Assessment" OR “elderly” OR “older-patient*” OR “older-person*” OR “older-people” OR “older-men” OR “older-women” OR “older-subject*” OR “older-adult*” OR “old-patient*” OR “old-person*” OR “old-people” OR “old-adult*” OR “geriatr*” OR “Parkinson Disease” OR “Parkinson diseas*” OR “stroke” OR “brain Ischemia” OR “intracranial hemorrhages” OR “Multiple Sclerosis” OR “Sarcopenia” OR “Muscular Atrophy” OR “sarcopenia*” OR “Hip Fractures” OR “femoral neck fracture*”)

AND

TI=("Exercise Therapy" OR "Motor Activity" OR "Physical Fitness" OR “strength-training” OR “resistance-training” OR “exercise-intervention*” OR “exercise” OR “vibration-training” OR “physical-exercise” OR “physical-activity” OR “aerobic-exercise”)

AND

TI=(“muscle quality” OR “specific tension” OR “echo intensity” OR “echogenicity” OR “intermuscular adipose tissue” OR “intermuscular fat” OR “intramuscular adipose tissue” OR “intramuscular fat” OR “muscle attenuation” OR “muscle density” OR “radiological density” OR “muscle composition” OR “muscle fat infiltration” OR “myosteatosis” OR “muscle architecture” AND ("Muscle Strength" OR “muscle-strength” OR “muscular-strength” OR "Muscle, Skeletal" OR “muscle*” OR “functional-capacity” OR “physical-function*” OR “muscle-thickness” OR “muscle-mass”)) OR AB=(“muscle quality” OR “specific tension” OR “echo intensity” OR “echogenicity” OR “intermuscular adipose tissue” OR “intermuscular fat” OR “intramuscular adipose tissue” OR “intramuscular fat” OR “muscle attenuation” OR “muscle density” OR “radiological density” OR “muscle composition” OR “muscle fat infiltration” OR “myosteatosis” OR “muscle architecture” AND ("Muscle Strength" OR “muscle-strength” OR “muscular-strength” OR "Muscle, Skeletal" OR “muscle*” OR “functional-capacity” OR “physical-function*” OR “muscle-thickness” OR “muscle-mass”))

AND

TI=("Randomized Controlled Trial” OR “random*” OR “randomized controlled trial” OR “controlled-study” OR “clinical-

**2. Online Supplementary Figures**

**Online Supplementary Figure S1.** Funnel plot for studies examining the effects of exercise interventions on morphological muscle quality.

**
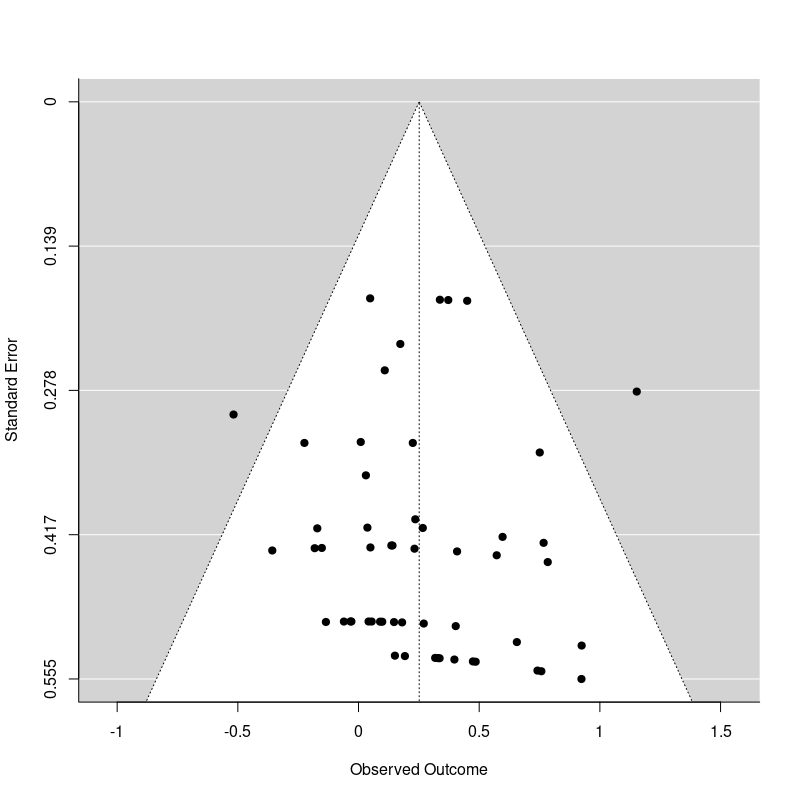
**

**Online Supplementary Figure S2.** Funnel plot for studies examining the effects of exercise interventions on neuromuscular muscle quality.


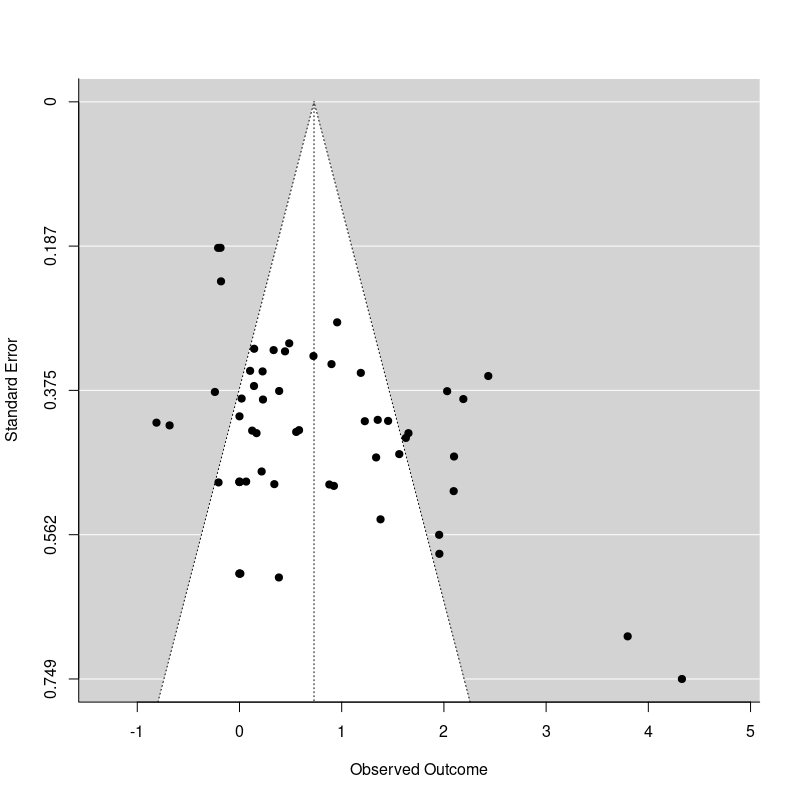


**Online Supplementary Figure S3.** Plot of influence diagnostics for studies examining the effects of exercise on morphological muscle quality. The six red symbols denote influential cases. The removal of these cases in the sensitivity analysis did not affect the overall effect found in the meta-analyses.

**Online Supplementary Figure S4.** Plot of influence diagnostics for studies examining the effects of exercise on neuromuscular muscle quality. The red symbol denotes an influential case. The removal of this case in the sensitivity analysis did not affect the overall effect found in the meta-analyses.****

**3. Online Supplementary Tables**

**Online Supplementary Table S1.** Characteristics of the studies included in the analyses.

Intervention _____ Session________ Functional Muscle quality_

Ref. Author Pop. Age, y n Type Exercise Dose Weeks  *n* Dur. Intens. Control outcomes Type Method Outcome

1 Bergamin HFOA 71 59 OT Aquatic, 60s exerc., 24 48 60 High Passive Mobility, MMQ DXA, Density land 30 rest strength pQCT fat area

2 Brightwell HFOA 73 23 AT Treadmill 70% of 24 72 45 High Passive Strength NMQ DXA Nm·kg^-1^

Walking HR reserve

3 Cadore LFOA 92 24 RT Machines 40-60% 12 24 40 Low Active Mobility, MMQ CT Density

1RM,8-10

reps

4 Coelho-Junior HFOA 67 26 RT Machines 1-4 sets, 26 52 40 Low Passive Mobility, NMQ DXA, kg/kg

12-15 reps strength, BIA

of 1RM power, bal.

5 Cunha ‘18 HFOA 69 62 RT Machines 1 or 3 sets, 12 36 15 Low Passive None NMQ DXA Kg/kg

10-15 reps

o1RM

6 Cunha ‘20 HFOA 69 62 RT Machines 1 or 3 sets, 12 36 15 Low Passive Strength NMQ DXA Kg/kg

10-15 reps

of 1RM

7 de Azevedo HFOA 66 36 RT Machines 2-4 sets 6-15 12 24 45 Low Active Strength MMQ US AU

Bach 6-15 of 1RM

8 Englund* LFOA 77 149 RT, Walking, RPE<15 24 72 60 Low Active Strength, MMQ CT Density, fat BT chair ex. ankle w. power NMQ area, kg/kg

9 Flor-Rufino LFOA 80 38 RT Machines 3 sets 10-15 24 39 65 High Passive Strength, MMQ MRI FA, MD

reps of >70% mobility

of RM

10 Fragala HFOA 70 23 RT Machines 3 sets of 6 12 90 Low Passive Strength NMQ DXA kg/kg

8-15 1RM

11 Ghasemikaram LFOA 78 43 RT Machines 1 set of 5-7 72 144 27 High Passive Strength NMQ MRI, N·kg^-1^

reps 65-80% DXA N·cm^-3^

of 1RM to

failure

12 Goodpaster LFOA 77 42 RT Machines Unknown 15 54 50 Low Passive Strength NMQ CT Nm·cm^-2^

AT Walking Unknown

13 Herda HFOA 67 65 OT El. Band 3 sets of 6 6 12 45 Low Passive Mobility, NMQ DXA Kg/kg

RT Dumb. Exerc. 8-12 strength

of 1RM

14 Hofmann* HFOA 84 91 RT El. band 2 sets of 24 48 60 Low Active None NMQ BIA Kg/kg

15 reps

15 Kargaran HFOA 68 24 OT Walking 20-min 8 24 20 Low Passive Mobility, NMQ DXA Kg/kg

±BFR walking power, bal.

16 Kennis HFOA 68 82 AT Diverse 60-min 52 156 60 Low Passive Strength, NMQ CT Nm·cm^-3^ RT power

Vibr.

17 Liao HFOA 67 56 RT El. Band RPE of 12 36 50 Low Passive Mobility, NMQ DXA Kg/kg

13/20 strength

18 Lopez HFOA 66 24 RT Machines 3 sets of 8 16 50 Low Active Mobility MMQ US AU,

8-15 reps strength NMQ N·m·mm^-1^

of 1RM

19 Markofski* HFOA 72 AT Treadmill 75% HR 24 72 50 Low Passive Mobility, NMQ DXA Nm·kg^-1^

Walking reserve strength

20 Oh HFOA 74 38 RT El. band 10-20 RM 18 36 60 Low Active Mobility, NMQ DXA Nm·kg^-1^

Zone strength

21 Osuka LFOA 72 78 RT El. bands 1-3 sets of 6 12 60 Low Passive None MMQ, US Nm/mm,

dumbbell, 8-10 reps NMQ AU

machines RPE 12-14

22 Pinto HFOA 66 36 RT Machines Concentric 6 12 40 High Passive Mobility NMQ US kg·mm^-1^

failure strength

23 Ribeiro HFOA 71 27 RT Machines 3 sets, 10-15 8 24 50 Low Active Strength NMQ DXA kg/kg

of 1RM

24 Scanlon HFOA 71 26 RT 2-4 sets RPE 5-6 6 12 40 Low Passive None MMQ DXA, Echo int.,

of 8-12 reps NMQ US Nm·kg^-1^

1RM

AT Walking

25 Sipilä HFOA 77 42 RT Air 60-75% 18 36 60 Low Passive None MMQ CT Fat area,

compr. of 1RM density

8-10 reps

26 Strasser* HFOA 82 54 RT El. band 15 RM 24 48 60 Low Active None NMQ DXA kg/kg

zone

27 Vojciechowski HFOA 69 46 OT Dance Exergaming 12 36 40 Low Passive Strength MMQ MRI Fat area

28 Wei HFOA 65 60 RT Machines 1-4 sets, 5- 24 48 60 Low Passive Strength MMQ pQCT Density fat 20 reps of area, fat density

66%1RM

29 Wilhelm HFOA 65 46 AT AT+RT 1-2 sets of 12 24 40 High Passive Mobility, MMQ US Echo int.

RT RT+AT 1RM+20-40 strength,

min cycling power

30 Yamada LFOA 84 112 RT El. band 3 sets x 12 24 40 Low Passive Mobility, MMQ BIA Echo int.

20 reps strength, US

bal., power

__________________________________________________________________________________________________________________________

Abbreviations in alphabetical order:

AT, aerobic training

BIA, Bioelectrical impedance analysis

CT, computed tomography

DXA, dual-energy X-ray absorptiometry

HFOA, higher functioning older adults

LFOA, lower functioning older adults

MMQ, morphological muscle quality

MRI, magnetic resonance imaging

NMQ, neuromuscular muscle quality

OT, other training

pQCT, peripheral quantitative computed tomography

RT, resistance training

US, ultrasound imaging

* intervention with dietary supplement

MOB, mobility

MQ, muscle quality

MRI, magnetic resonance imaging

MS/P, muscle strength, power

MT, multimodal training

MT, muscle thickness

MV, muscle volume

MVC, maximal voluntary contraction force or torque

NMQ, neuromuscular muscle quality

OEI, other exercise intervention

P, passive control group

PF, ankle plantarfelxors

pQCT, peripheral quantitative computed tomography

RT, resistance training

S,min, session duration

S,n, session number

Suppl., Supplement

TR, trunk muscles

TUG, timed up and go test

US, ultrasound imaging

WF, wrist flexors

**References**

1 Bergamin M, Ermolao A, Tolomio S, Berton L, Sergi G, Zaccaria M. Water- versus land-based exercise in elderly subjects: effects on physical performance and body composition. Clin Interv Aging. 2013;8:1109-17.

2 Brightwell CR, Markofski MM, Moro T, Fry CS, Porter C, Volpi E, et al. Moderate-intensity aerobic exercise improves skeletal muscle quality in older adults. Transl Sports Med. 2019 Apr;2(3):109-19.

3 Cadore EL, Casas-Herrero A, Zambom-Ferraresi F, Idoate F, Millor N, Gomez M, et al. Multicomponent exercises including muscle power training enhance muscle mass, power output, and functional outcomes in institutionalized frail nonagenarians. Age (Dordr). 2014 Apr;36(2):773-85.

4 Coelho-Junior HJ, de Oliveira Goncalvez I, Sampaio RAC, Sewo Sampaio PY, Cadore EL, Izquierdo M, et al. Periodized and non-periodized resistance training programs on body composition and physical function of older women. Exp Gerontol. 2019 Jul 1;121:10-8.

5 Cunha PM, Nunes JP, Tomeleri CM, Nascimento MA, Schoenfeld BJ, Antunes M, et al. Resistance Training Performed With Single and Multiple Sets Induces Similar Improvements in Muscular Strength, Muscle Mass, Muscle Quality, and IGF-1 in Older Women: A Randomized Controlled Trial. J Strength Cond Res. 2020 Apr;34(4):1008-16.

6 Cunha PM, Tomeleri CM, Nascimento MAD, Nunes JP, Antunes M, Nabuco HCG, et al. Improvement of cellular health indicators and muscle quality in older women with different resistance training volumes. J Sports Sci. 2018 Dec;36(24):2843-8.

7 de Azevedo Bach S, Radaelli R, Beck Schemes M, Neske R, Garbelotto C, Roschel H, et al. Can supplemental protein to low-protein containing meals superimpose on resistance-training muscle adaptations in older adults? A randomized clinical trial. Exp Gerontol. 2022 Jun 1;162:111760.

8 Englund DA, Kirn DR, Koochek A, Zhu H, Travison TG, Reid KF, et al. Nutritional Supplementation With Physical Activity Improves Muscle Composition in Mobility-Limited Older Adults, The VIVE2 Study: A Randomized, Double-Blind, Placebo-Controlled Trial. J Gerontol A Biol Sci Med Sci. 2017 Dec 12;73(1):95-101.

9 Flor-Rufino C, Barrachina-Igual J, Perez-Ros P, Pablos-Monzo A, Sanz-Requena R, Martinez-Arnau FM. Fat infiltration and muscle hydration improve after high-intensity resistance training in women with sarcopenia. A randomized clinical trial. Maturitas. 2023 Feb;168:29-36.

10 Fragala MS, Jajtner AR, Beyer KS, Townsend JR, Emerson NS, Scanlon TC, et al. Biomarkers of muscle quality: N-terminal propeptide of type III procollagen and C-terminal agrin fragment responses to resistance exercise training in older adults. J Cachexia Sarcopenia Muscle. 2014 Jun;5(2):139-48.

11 Ghasemikaram M, Engelke K, Kohl M, von Stengel S, Kemmler W. Detraining Effects on Muscle Quality in Older Men with Osteosarcopenia. Follow-Up of the Randomized Controlled Franconian Osteopenia and Sarcopenia Trial (FrOST). Nutrients. 2021 May 1;13(5).

12 Goodpaster BH, Chomentowski P, Ward BK, Rossi A, Glynn NW, Delmonico MJ, et al. Effects of physical activity on strength and skeletal muscle fat infiltration in older adults: a randomized controlled trial. J Appl Physiol (1985). 2008 Nov;105(5):1498-503.

13 Herda AA, Nabavizadeh O. Short-term resistance training in older adults improves muscle quality: A randomized control trial. Exp Gerontol. 2021 Mar;145:111195.

14 Hofmann M, Schober-Halper B, Oesen S, Franzke B, Tschan H, Bachl N, et al. Effects of elastic band resistance training and nutritional supplementation on muscle quality and circulating muscle growth and degradation factors of institutionalized elderly women: the Vienna Active Ageing Study (VAAS). Eur J Appl Physiol. 2016 May;116(5):885-97.

15 Kargaran A, Abedinpour A, Saadatmehr Z, Yaali R, Amani-Shalamzari S, Gahreman D. Effects of dual-task training with blood flow restriction on cognitive functions, muscle quality, and circulatory biomarkers in elderly women. Physiol Behav. 2021 Oct 1;239:113500.

16 Kennis E, Verschueren SM, Bogaerts A, Coudyzer W, Boonen S, Delecluse C. Effects of fitness and vibration training on muscle quality: a 1-year postintervention follow-up in older men. Arch Phys Med Rehabil. 2013 May;94(5):910-8.

17 Liao CD, Tsauo JY, Huang SW, Ku JW, Hsiao DJ, Liou TH. Effects of elastic band exercise on lean mass and physical capacity in older women with sarcopenic obesity: A randomized controlled trial. Sci Rep. 2018 Feb 2;8(1):2317.

18 Lopez P, Crosby BJ, Robetti BP, Turella DJP, Weber TAS, de Oliveira ML, et al. Effects of an 8-week resistance training intervention on plantar flexor muscle quality and functional capacity in older women: A randomised controlled trial. Exp Gerontol. 2020 Sep;138:111003.

19 Markofski MM, Jennings K, Timmerman KL, Dickinson JM, Fry CS, Borack MS, et al. Effect of Aerobic Exercise Training and Essential Amino Acid Supplementation for 24 Weeks on Physical Function, Body Composition, and Muscle Metabolism in Healthy, Independent Older Adults: A Randomized Clinical Trial. J Gerontol A Biol Sci Med Sci. 2019 Sep 15;74(10):1598-604.

20 Oh SL, Kim HJ, Woo S, Cho BL, Song M, Park YH, et al. Effects of an integrated health education and elastic band resistance training program on physical function and muscle strength in community-dwelling elderly women: Healthy Aging and Happy Aging II study. Geriatr Gerontol Int. 2017 May;17(5):825-33.

21 Osuka Y, Kojima N, Nishihara K, Sasai H, Wakaba K, Tanaka K, et al. beta-Hydroxy-beta-Methylbutyrate Supplementation May Not Enhance Additional Effects of Exercise on Muscle Quality in Older Women. Med Sci Sports Exerc. 2022 Apr 1;54(4):543-50.

22 Pinto RS, Correa CS, Radaelli R, Cadore EL, Brown LE, Bottaro M. Short-term strength training improves muscle quality and functional capacity of elderly women. Age (Dordr). 2014 Feb;36(1):365-72.

23 Ribeiro AS, Picoloto A, Nunes JP, Bezerra ES, Schoenfeld BJ, Cyrino ES. Effects of Different Resistance Training Loads on the Muscle Quality Index in Older Women. J Strength Cond Res. 2022 May 1;36(5):1445-9.

24 Scanlon TC, Fragala MS, Stout JR, Emerson NS, Beyer KS, Oliveira LP, et al. Muscle architecture and strength: adaptations to short-term resistance training in older adults. Muscle Nerve. 2014 Apr;49(4):584-92.

25 Sipila S, Suominen H. Effects of strength and endurance training on thigh and leg muscle mass and composition in elderly women. J Appl Physiol (1985). 1995 Jan;78(1):334-40.

26 Strasser EM, Hofmann M, Franzke B, Schober-Halper B, Oesen S, Jandrasits W, et al. Strength training increases skeletal muscle quality but not muscle mass in old institutionalized adults: a randomized, multi-arm parallel and controlled intervention study. Eur J Phys Rehabil Med. 2018 Dec;54(6):921-33.

27 Vojciechowski AS, Silva CTS, Rodrigues EV, Gallo LH, Melo Filho J, Gomes ARS. Does Physical Dance Training with Virtual Games Change Muscle Quality of Community-Dwelling Older Women? Games Health J. 2021 Dec;10(6):391-9.

28 Wei M, Meng D, Guo H, He S, Tian Z, Wang Z, et al. Hybrid Exercise Program for Sarcopenia in Older Adults: The Effectiveness of Explainable Artificial Intelligence-Based Clinical Assistance in Assessing Skeletal Muscle Area. Int J Environ Res Public Health. 2022 Aug 12;19(16).

29 Wilhelm EN, Rech A, Minozzo F, Botton CE, Radaelli R, Teixeira BC, et al. Concurrent strength and endurance training exercise sequence does not affect neuromuscular adaptations in older men. Exp Gerontol. 2014 Dec;60:207-14.

30 Yamada M, Kimura Y, Ishiyama D, Nishio N, Otobe Y, Tanaka T, et al. Synergistic effect of bodyweight resistance exercise and protein supplementation on skeletal muscle in sarcopenic or dynapenic older adults. Geriatr Gerontol Int. 2019 May;19(5):429-37.

**Online Supplementary Table S2.** Multi-variable meta-regression model for neuromuscular muscle quality. For morphological muscle quality, there were no significant moderators of the exercise effects because heterogeneity was zero.

**Neuromuscular muscle quality**

**Model: ES ~ Modality + Intensity + Method**

Number of studies = 26

Number of outcomes = 55 (min = 1 , mean = 2.12 , median = 2 , max = 8 )

Rho = 0.8

I^2^= 65.8677

T^2^ = 0.290817

Estimate StdErr t-value dfs P(|t|>) 95% CI.L 95% CI.U Sig

1 X.Intercept. -0.770 0.376 -2.05 6.42 0.08329 -1.6752 0.1352 *

2 ModalityRT 1.161 0.258 4.50 7.29 0.00252 0.5563 1.7662 ***

3 IntensityHI 0.523 0.150 3.49 2.91 0.04153 0.0384 1.0069 **

4 MethodCT 1.025 0.418 2.45 9.07 0.03629 0.0815 1.9687 **

5 MethodDXA 0.969 0.366 2.65 3.37 0.06809 -0.1254 2.0627 *

6 MethodMRI 1.118 0.312 3.59 4.89 0.01638 0.3112 1.9244 **

7 MethodUS -0.746 0.298 -2.50 4.44 0.06030 -1.5422 0.0495 *

Significance codes: < .01 *** < .05 ** < .10 *

Note: If df < 4, do not trust the results

Reference group:

Other Exercise Intervention (OEI) (for Modality)

Low Intensity (LI) (for Intensity)

Bioelectrical impedance (BIA) (for Methods of measuring muscle quality)

Test of significance of the "Method" variable as a whole:

Test Fstat df_num df_denom p_val sig

HTZ 36.6 4 4.4 <0.001 ***

The number of studies according to the intervention modality: OEI: 6; RT: 15

The number of studies according to Intensity: HI: 3 LI: 18

The number of studies using individual methods: BIA: 2; CT: 3; DXA: 13; MRI: 1; US: 4

**Online Supplementary Table S3.** Individual risk of bias assessment for studies examining morphological and neuromuscular muscle quality outcomes.

| **Studies** | **Randomisation process** | **Deviations from intended interventions** | **Missing outcome data** | **Measurements of the outcome** | **Selection of the reported results** | **Overall bias** |
| --- | --- | --- | --- | --- | --- | --- |
| 1. Bergamin et al., 2013 | Some concerns | Low | Low | Low | Low | Some concerns |
| 2. Brightwell et al., 2019 | Some concerns | Low | Low | Some concerns | Low | Some concerns |
| 3. Cadore et al., 2014 | Some concerns | Low | Low | Low | Low | Some concerns |
| 4. Coelho-Junior et al., 2019 | Some concerns | Low | Low | Low | Low | Some concerns |
| 5. Cunha et al., 2018 | Some concerns | Low | Low | High | Low | High |
| 6. Cunha et al., 2020 | Some concerns | Low | Low | Hight | Low | High |
| 7. de Azevedo Bach et al., 2022 | Low | Low | Low | Low | Some concerns | Some concerns |
| 8. Englund et al., 2018 | Low | Low | Low | Low | High | Some concerns |
| 9. Flor-Rufino et al., 2023 | Low | Low | Low | Low | Some concerns | Some concerns |
| 10. Fragala et al., 2014 | High | Low | Low | High | Low | High |
| 11. Ghasemikaram et al., 2021 | Some concerns | Low | Low | Low | Low | Some concerns |
| 12. Goodpaster et al., 2008 | Some concerns | Low | Low | Low | Low | Some concerns |
| 13. Herda et al., 2021 | Some concerns | Low | Low | Low | High | Some concerns |
| 14. Hofmann et al., 2016 | Some concerns | Low | Low | Low | High | Some concerns |
| 15. Kargaran et al., 2021 | High | Low | Low | Low | Some concerns | High |
| 16. Kennis et al., 2013 | Some concerns | Low | Low | Low | Low | Some concerns |
| 17. Liao et al., 2018 | Some concerns | Low | Low | Low | Low | Some concerns |
| 18. Lopez et al., 2020 | Low | Low | Low | Low | Low | Low |
| 19. Markofski et al., 2019 | Low | Low | Low | Low | High | Some concerns |
| 20. Oh et al., 2017 | Some concerns | Low | Low | High | Low | High |
| 21. Osuka et al., 2022 | Low | Low | Low | Low | Some concerns | Some concerns |
| 22. Pinto et al., 2014 | Some concerns | Low | Low | Low | Low | Some concerns |
| 23. Ribeiro et al., 2022 | Low | Low | Low | Low | Some concerns | Some concerns |
| 24. Scanlon et al., 2014 | High | Low | Low | High | Low | High |
| 25. Strasser et al., 2019 | High | Low | High | Low | Low | High |
| 26. Sipilä et al., 1995 | High | Low | Low | Low | High | Some concerns |
| 27. Vojciechowski et al., 2021 | Some concerns | Low | Low | Low | Low | Some concerns |
| 28. Wei et al., 2022 | Low | Low | Low | Low | Some concerns | Some concerns |
| 29. Wilhelm et al., 2014 | Some concerns | Low | Low | High | Low | High |
| 30. Yamada et al., 2019 | Some concerns | Low | Low | Low | Low | Some concerns |
|  |  |  |  |  |  |  |

**References**

1 Bergamin M, Ermolao A, Tolomio S, Berton L, Sergi G, Zaccaria M. Water- versus land-based exercise in elderly subjects: effects on physical performance and body composition. Clin Interv Aging. 2013;8:1109-17.

2 Brightwell CR, Markofski MM, Moro T, Fry CS, Porter C, Volpi E, et al. Moderate-intensity aerobic exercise improves skeletal muscle quality in older adults. Transl Sports Med. 2019 Apr;2(3):109-19.

3 Cadore EL, Casas-Herrero A, Zambom-Ferraresi F, Idoate F, Millor N, Gomez M, et al. Multicomponent exercises including muscle power training enhance muscle mass, power output, and functional outcomes in institutionalized frail nonagenarians. Age (Dordr). 2014 Apr;36(2):773-85.

4 Coelho-Junior HJ, de Oliveira Goncalvez I, Sampaio RAC, Sewo Sampaio PY, Cadore EL, Izquierdo M, et al. Periodized and non-periodized resistance training programs on body composition and physical function of older women. Exp Gerontol. 2019 Jul 1;121:10-8.

5 Cunha PM, Nunes JP, Tomeleri CM, Nascimento MA, Schoenfeld BJ, Antunes M, et al. Resistance Training Performed With Single and Multiple Sets Induces Similar Improvements in Muscular Strength, Muscle Mass, Muscle Quality, and IGF-1 in Older Women: A Randomized Controlled Trial. J Strength Cond Res. 2020 Apr;34(4):1008-16.

6 Cunha PM, Tomeleri CM, Nascimento MAD, Nunes JP, Antunes M, Nabuco HCG, et al. Improvement of cellular health indicators and muscle quality in older women with different resistance training volumes. J Sports Sci. 2018 Dec;36(24):2843-8.

7 de Azevedo Bach S, Radaelli R, Beck Schemes M, Neske R, Garbelotto C, Roschel H, et al. Can supplemental protein to low-protein containing meals superimpose on resistance-training muscle adaptations in older adults? A randomized clinical trial. Exp Gerontol. 2022 Jun 1;162:111760.

8 Englund DA, Kirn DR, Koochek A, Zhu H, Travison TG, Reid KF, et al. Nutritional Supplementation With Physical Activity Improves Muscle Composition in Mobility-Limited Older Adults, The VIVE2 Study: A Randomized, Double-Blind, Placebo-Controlled Trial. J Gerontol A Biol Sci Med Sci. 2017 Dec 12;73(1):95-101.

9 Flor-Rufino C, Barrachina-Igual J, Perez-Ros P, Pablos-Monzo A, Sanz-Requena R, Martinez-Arnau FM. Fat infiltration and muscle hydration improve after high-intensity resistance training in women with sarcopenia. A randomized clinical trial. Maturitas. 2023 Feb;168:29-36.

10 Fragala MS, Jajtner AR, Beyer KS, Townsend JR, Emerson NS, Scanlon TC, et al. Biomarkers of muscle quality: N-terminal propeptide of type III procollagen and C-terminal agrin fragment responses to resistance exercise training in older adults. J Cachexia Sarcopenia Muscle. 2014 Jun;5(2):139-48.

11 Ghasemikaram M, Engelke K, Kohl M, von Stengel S, Kemmler W. Detraining Effects on Muscle Quality in Older Men with Osteosarcopenia. Follow-Up of the Randomized Controlled Franconian Osteopenia and Sarcopenia Trial (FrOST). Nutrients. 2021 May 1;13(5).

12 Goodpaster BH, Chomentowski P, Ward BK, Rossi A, Glynn NW, Delmonico MJ, et al. Effects of physical activity on strength and skeletal muscle fat infiltration in older adults: a randomized controlled trial. J Appl Physiol (1985). 2008 Nov;105(5):1498-503.

13 Herda AA, Nabavizadeh O. Short-term resistance training in older adults improves muscle quality: A randomized control trial. Exp Gerontol. 2021 Mar;145:111195.

14 Hofmann M, Schober-Halper B, Oesen S, Franzke B, Tschan H, Bachl N, et al. Effects of elastic band resistance training and nutritional supplementation on muscle quality and circulating muscle growth and degradation factors of institutionalized elderly women: the Vienna Active Ageing Study (VAAS). Eur J Appl Physiol. 2016 May;116(5):885-97.

15 Kargaran A, Abedinpour A, Saadatmehr Z, Yaali R, Amani-Shalamzari S, Gahreman D. Effects of dual-task training with blood flow restriction on cognitive functions, muscle quality, and circulatory biomarkers in elderly women. Physiol Behav. 2021 Oct 1;239:113500.

16 Kennis E, Verschueren SM, Bogaerts A, Coudyzer W, Boonen S, Delecluse C. Effects of fitness and vibration training on muscle quality: a 1-year postintervention follow-up in older men. Arch Phys Med Rehabil. 2013 May;94(5):910-8.

17 Liao CD, Tsauo JY, Huang SW, Ku JW, Hsiao DJ, Liou TH. Effects of elastic band exercise on lean mass and physical capacity in older women with sarcopenic obesity: A randomized controlled trial. Sci Rep. 2018 Feb 2;8(1):2317.

18 Lopez P, Crosby BJ, Robetti BP, Turella DJP, Weber TAS, de Oliveira ML, et al. Effects of an 8-week resistance training intervention on plantar flexor muscle quality and functional capacity in older women: A randomised controlled trial. Exp Gerontol. 2020 Sep;138:111003.

19 Markofski MM, Jennings K, Timmerman KL, Dickinson JM, Fry CS, Borack MS, et al. Effect of Aerobic Exercise Training and Essential Amino Acid Supplementation for 24 Weeks on Physical Function, Body Composition, and Muscle Metabolism in Healthy, Independent Older Adults: A Randomized Clinical Trial. J Gerontol A Biol Sci Med Sci. 2019 Sep 15;74(10):1598-604.

20 Oh SL, Kim HJ, Woo S, Cho BL, Song M, Park YH, et al. Effects of an integrated health education and elastic band resistance training program on physical function and muscle strength in community-dwelling elderly women: Healthy Aging and Happy Aging II study. Geriatr Gerontol Int. 2017 May;17(5):825-33.

21 Osuka Y, Kojima N, Nishihara K, Sasai H, Wakaba K, Tanaka K, et al. beta-Hydroxy-beta-Methylbutyrate Supplementation May Not Enhance Additional Effects of Exercise on Muscle Quality in Older Women. Med Sci Sports Exerc. 2022 Apr 1;54(4):543-50.

22 Pinto RS, Correa CS, Radaelli R, Cadore EL, Brown LE, Bottaro M. Short-term strength training improves muscle quality and functional capacity of elderly women. Age (Dordr). 2014 Feb;36(1):365-72.

23 Ribeiro AS, Picoloto A, Nunes JP, Bezerra ES, Schoenfeld BJ, Cyrino ES. Effects of Different Resistance Training Loads on the Muscle Quality Index in Older Women. J Strength Cond Res. 2022 May 1;36(5):1445-9.

24 Scanlon TC, Fragala MS, Stout JR, Emerson NS, Beyer KS, Oliveira LP, et al. Muscle architecture and strength: adaptations to short-term resistance training in older adults. Muscle Nerve. 2014 Apr;49(4):584-92.

25 Sipila S, Suominen H. Effects of strength and endurance training on thigh and leg muscle mass and composition in elderly women. J Appl Physiol (1985). 1995 Jan;78(1):334-40.

26 Strasser EM, Hofmann M, Franzke B, Schober-Halper B, Oesen S, Jandrasits W, et al. Strength training increases skeletal muscle quality but not muscle mass in old institutionalized adults: a randomized, multi-arm parallel and controlled intervention study. Eur J Phys Rehabil Med. 2018 Dec;54(6):921-33.

27 Vojciechowski AS, Silva CTS, Rodrigues EV, Gallo LH, Melo Filho J, Gomes ARS. Does Physical Dance Training with Virtual Games Change Muscle Quality of Community-Dwelling Older Women? Games Health J. 2021 Dec;10(6):391-9.

28 Wei M, Meng D, Guo H, He S, Tian Z, Wang Z, et al. Hybrid Exercise Program for Sarcopenia in Older Adults: The Effectiveness of Explainable Artificial Intelligence-Based Clinical Assistance in Assessing Skeletal Muscle Area. Int J Environ Res Public Health. 2022 Aug 12;19(16).

29 Wilhelm EN, Rech A, Minozzo F, Botton CE, Radaelli R, Teixeira BC, et al. Concurrent strength and endurance training exercise sequence does not affect neuromuscular adaptations in older men. Exp Gerontol. 2014 Dec;60:207-14.

30 Yamada M, Kimura Y, Ishiyama D, Nishio N, Otobe Y, Tanaka T, et al. Synergistic effect of bodyweight resistance

exercise and protein supplementation on skeletal muscle in sarcopenic or dynapenic older adults. Geriatr Gerontol

Int. 2019 May;19(5):429-37.
